# Assessing the reliability of paleomagnetic datasets using the R package PmagDiR

**DOI:** 10.1038/s41598-024-52001-x

**Published:** 2024-01-18

**Authors:** Edoardo Dallanave

**Affiliations:** Faculty of Geosciences, University of Bremen, Bremen, Germany

**Keywords:** Palaeomagnetism, Software

## Abstract

Paleomagnetism is the most important source of information for determining the position of tectonic plates in the geological past. Over the last few decades significant advancements have been made in improving the reliability of paleomagnetic data, ranging from analytical methods to statistical assessments. Here I present the first version of *PmagDiR*, an R-based open-source package which displays, assesses the reliability, and, when possible, corrects given paleomagnetic directions distributions. The main functions of the package are to: plot paleomagnetic directions, their averages and confidence boundaries; apply different direction cut-offs to identify and filter outliers; perform a test for antipodality; compare the distribution shape with the one predicted by a widely accepted paleosecular variation model; correct flawed distributions for paleomagnetic inclination flattening; and correct for strain-derived paleomagnetic directions deviations when the strain fabric is known. Furthermore, directions can be converted in virtual geomagnetic poles and plotted on a spherical projection for comparison with reference apparent polar wander paths. All main functions of the *PmagDiR* package return results as comma separated value text files as well as vector graphic files (pdf), optimized for publication purpose with minimal manipulation.

## Introduction

Paleomagnetic data are the most important source of information for paleogeographic reconstructions. This is because paleomagnetism is the only geophysical tool that allows anchoring the position of the tectonic plates with respect to the Earth’s spin axis in the geological past^1–3^. Accurate paleogeography is essential not only for geodynamic problems per se, but also for understanding past climate dynamics^4^, paleogeographic distributions of fossils^5^ and for absolute positions of land and ocean in order to constrain paleoclimate numerical models^6,7^. Paleomagnetic directions are the foundation of all paleomagnetic studies. Statistical approaches for paleomagnetic directions analysis went through significant advancements in the last two decades, and they are currently under constant development. Improvements range from the analysis of single paleomagnetic directions^8^, the analysis of direction clusters^9–12^, and the determination of paleomagnetic poles^13^ and their combinations required to build an apparent polar wander path (APWP)^14–16^.

Generally speaking, scientific data handling, calculations, and statistical analyses are strongly supported by specific software. Several software packages for the analysis of paleomagnetic directions, direction clusters and paleomagnetic poles have been developed through the years. These include widely used packages like PaleoMac^17^, Remasoft^18^, PuffinPlot^19^, Paleomagnetism.org^20,21^, and PmagPy^22^. Among the different programming languages used in geosciences, R (https://www.r-project.org/) has numerous applications in many Earth science disciplines^23–26^. Here I present *PmagDiR*, an R-based toolbox for displaying, assessing reliability, and, when possible, correcting a paleomagnetic directions distribution.

## Why PmagDiR?

The open-source package *PmagDiR*, in its current and future versions, is archived on GitHub (https://github.com/edoardo-paleomag/PmagDiR) and it is optimized for RStudio Desktop. RStudio Desktop (https://posit.co/download/rstudio-desktop/) is a user-friendly interface developed to facilitate the compilation and usage of computational and graphical codes in the R language.

*PmagDiR* allows the user to (1) plot paleomagnetic directions on an equal area projection with associated average directions and confidence boundaries^27^, (2) apply directions cut-off based on virtual geomagnetic poles (VGPs) distance from the average pole^28,29^, (3) perform a bootstrap-based test for antipodality^30^, (4) compare the paleomagnetic directions distribution shape with the one predicted by TK03.GAD paleosecular variations model of Tauxe and Kent (2004)^9^, (5) correct inclination flattening if present, (6) correct strain-derived paleomagnetic directions deviation, in the case of known fabric^31–33^, and (7) plot VGPs distributions and averages on a spherical projection for comparison with reference data.

Within these computational codes, *PmagDiR* offers some tools that are, to date, unique. These are (as detailed below): the directions *dynamic cut-off*, which incorporates the correction of inclination shallowing of paleomagnetic directions within the iterative filtering process; the possibility of assessing the directions distribution shape (before application of any correcting protocol) with bootstrapped-derived confidence boundaries; and the “unstrain” process of paleomagnetic directions derived from deformed rocks with known magnetic fabric. Every function of *PmagDiR* is supported by a Help Document (with examples) describing the functionalities and options available. Results are exported into the working directory as comma separated value (csv) text files as well as vector graphic files (pdf) optimized for publication purpose with minimal manipulation.

Two reference (real) paleomagnetic datasets are provided within the package to help the user get familiar with the different functionalities, and they are used to describe a typical workflow in the supporting information available online. The first set (*Ardo_PmagDiR*) consists of paleomagnetic directions from Paleocene sedimentary rocks exposed in the Venetian Alps of Italy^34^. The second (*Km_PmagDiR*) includes paleomagnetic directions from the early Eocene sedimentary record exposed near Koumac in New Caledonia^31^ and the average anisotropy of magnetic susceptibility (AMS) tensor from the same record (*Km_AMS)*.

In the following paragraphs I present the main functionalities of *PmagDiR* and their theoretical background*,* while mathematical details are explained in the Supplementary File.

## Plotting directions, their averages, and confidence angles

*PmagDiR* allows plotting paleomagnetic directions with user-defined symbols and colors. Multiple datasets can be plotted on the same equal area diagram. In case of bimodal distributions, directions can be plotted either in the original two-modes or automatically “flipped” toward a common (down or up) pointing mode. The distinction between one or two modes is based on the angular distance from the maximum variance eigenvector of the orientation matrix^35^. This process (detailed in the computational background available as Supplementary File) is particularly relevant as it is used by all scripts of *PmagDiR* that automatically separate direction modes.

The average direction(s) of a set is calculated by applying the standard cartesian approach of Fisher^27^. The confidence area can be estimated by applying two strategies. The first is by using the standard Fisher^27^ 95% half-angle of confidence, as conventionally done in most publications. However, the standard Fisher 95% cone of confidence can often be regarded as unrealistic. This is because an ideal Fisher distribution is characterized by (1) a uniform distribution of directions around the average and (2) an exponential decay of directions occurrence with the radial distance from the average^36^, conditions that are often not matched by natural sets. An alternative way is to first calculate the distribution of VGPs from the directions. A VGP is defined as the point on the Earth’s surface where the imaginary pole resulting from the measured declination and inclination is located. Geomagnetic paleosecular variations models^38^ show that the distribution of VGPs, rather than paleomagnetic directions, is expected to be circularly distributed around the average (although not necessarily respecting condition (2) described above). A confidence ellipse around the average paleomagnetic direction can therefore be derived from the 95% confidence cone around the paleomagnetic pole (i.e., mean of the VGPs) by using the equations proposed by Deenen et al. (2011^38^; see Supplementary File).

**Figure 1:**
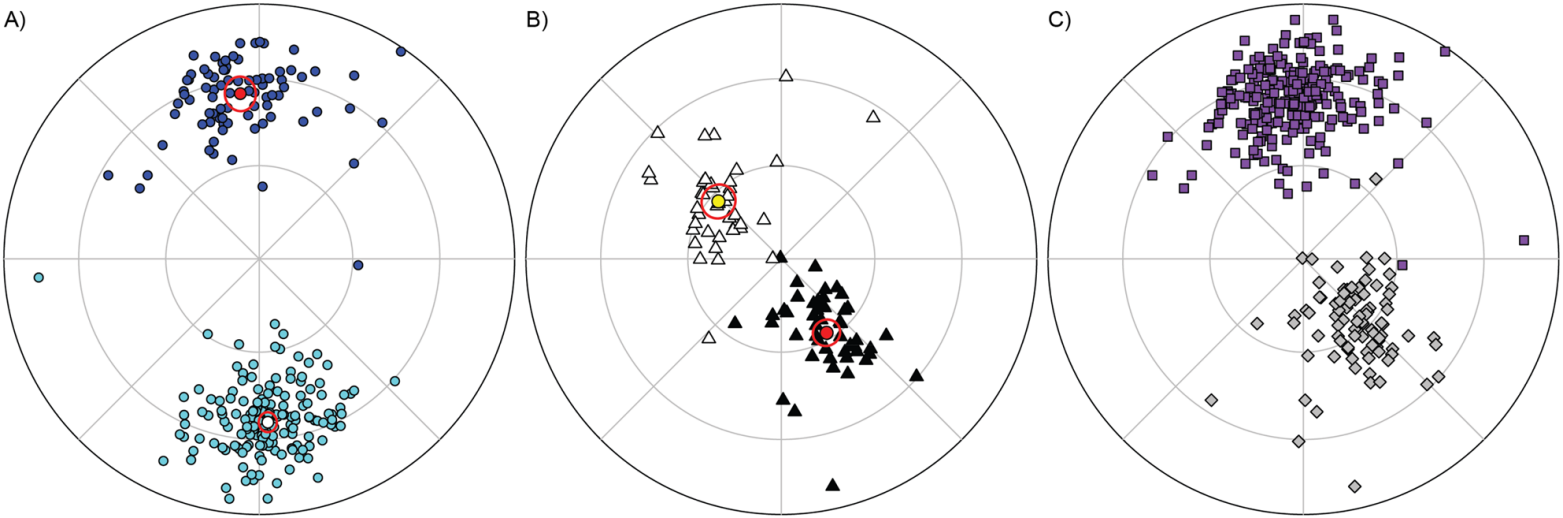
Examples of equal area diagrams plotted with PmagDiR::plot_DI; a choice of symbols and colors can be selected by the user to plot multiple datasets on the same diagram. Details about the “plot_DI” (and all functions of PmagDiR) functionalities are accessible by typing “?plot_DI” in the RStudio console. (**A**) Ardo_PmagDiR paleomagnetic directions dataset^34^ with average directions and 95% confidence ellipses calculated with the function “ellips_plot”. (**B**) Km_PmagDiR paleomagnetic dataset^39^ with average directions and Fisher 95% confidence calculated with the function “fisher_plot”. (**C**) Both datasets of (**A**) and (**B**) plotted on a common down-pointing mode.

By using *PmagDiR*, the average direction(s) and the confidence ellipses are plotted either on an existing or on a new equal area diagram. Statistical parameters of bimodal distributions are automatically calculated for both modes, as well as for the whole dataset converted to a common direction. Results are shown in the R-Studio console and exported as a text file (unless indicated otherwise in the command line), as well as a vector (pdf) file (Fig. 1).

## Reliability of paleomagnetic directions

### Paleomagnetic directions dynamic cut-off

Paleomagnetic datasets often include directions acquired during both normal and reversed polarity (bimodal distributions), as well as transitional directions. Transitional directions typically characterize geomagnetic reversals or geomagnetic excursions, which represent specific and relatively brief events when the intensity of the field collapses. During these events VGPs depart from what is expected from the normal geomagnetic secular variation area of the globe^40–43^. When using paleomagnetic data for paleogeographic reconstructions, it is common practice to filter a given dataset for its transitional directions. In early works such a filter was applied by adopting a fixed VGP cut-off angle from the average paleomagnetic pole of typically 40° or 45°, where directions with associated VGP exceeding that angular distance were considered transitional^29,44,45^. Vandamme (1994)^28^ proposed a more sophisticated filtering protocol based on the VGPs scatter, which applies a recursive method to determine the most appropriate cut-off angle. Despite widely used, Tauxe et al. (2008)^46^ suggested that the application of directions cut-off should not be applied before comparison with paleosecular variations model such as the TK03.GAD, like it is done to detect and correct for inclination shallowing (see below). This problem was extensively explored by Vaes et al. (2021)^10^, who also advise against cut-off application before inclination flattening correction, because it may lead to underestimation of the inclination flattening degree. Nonetheless cut-off is still commonly applied, also because “transitional” directions do not necessarily represent only real geomagnetic field events, but they can be the consequence of local remagnetization or sedimentological phenomenon like intra-strata slumping^47^.

**Figure 2:**
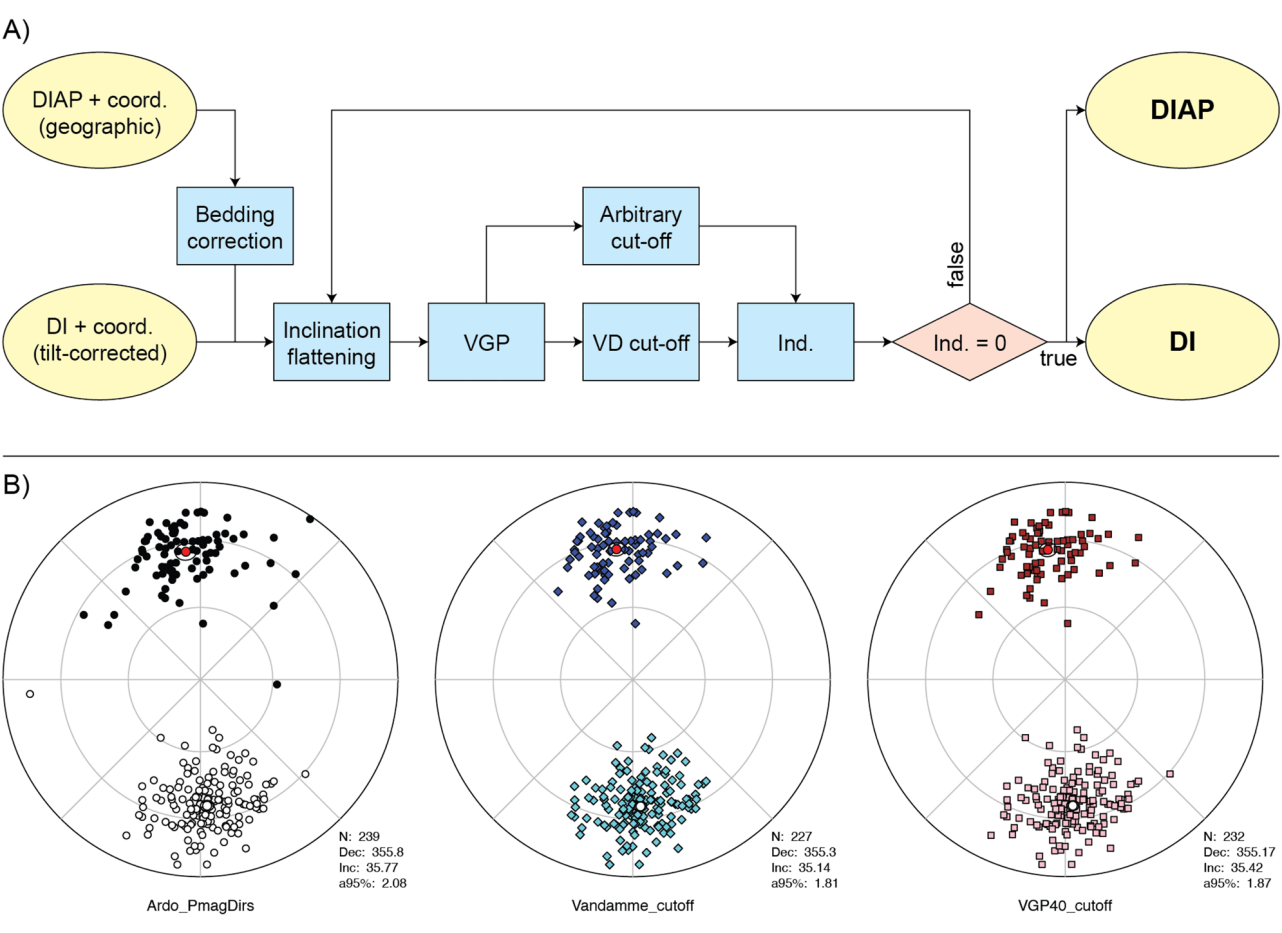
Dynamic cut-off of paleomagnetic transitional directions. (**A**) Block-model of the PmagDiR::cut_DI algorithm. Input file contains either declination and inclination (DI) of directions already corrected for bedding tilt, as well as bedding-dip azimuth and plunge (DIAP) if directions are provided in geographic coordinates. The coordinates of the sampling site (coord.) are specified in the console when calling the function (see PmagDiR::cut_DI documentation). Inclination flattening is estimated through the distribution elongation method^9^. Virtual geomagnetic poles (VGPs) are calculated and averaged to obtain the paleomagnetic pole, then the cut-off angle is defined either as proposed by Vandamme^28^ (VD, default option) or defined arbitrarily. Directions associated with the VGPs defined as transitional by the selected cut-off are indexed (Ind.), and excluded from the original dataset before repeating the process. When no more directions are excluded from the cut-off (Ind. = 0 is true) the function returns the list of filtered directions in the same form as the entry file. (**B**) Application of the Vandamme^28^ and a fixed 40° dynamic cut-off to the Ardo_PmagDiR dataset; in all diagrams: *N* number of directions, *Dec* average declination, *Inc* average inclination, *a95* Fisher^27^ angle of confidence.

**Figure 3:**
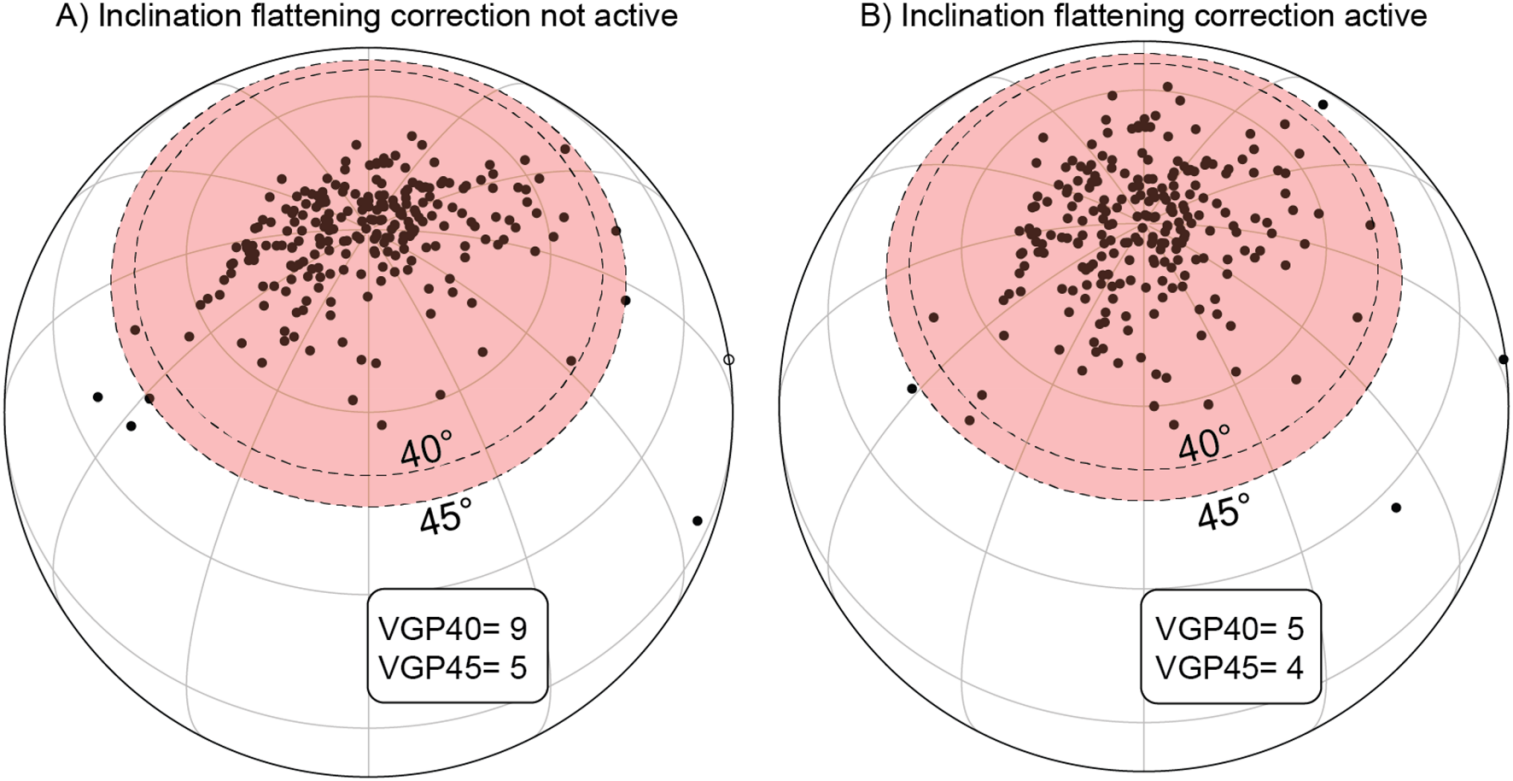
Effect of the inclination flattening correction on two fixed angle cut-offs based on the virtual geomagnetic pole (VGP) distance from the mean paleomagnetic poles. The VGP distributions ((**A**) is without inclination flattening correction, while (**B**) is with inclination flattening correction) have been rotated by placing the average position on the North pole. The numbers within the insets indicate the directions eliminated by the two cut-off angles.

The *PmagDiR* package gives the opportunity to apply a *dynamic* cut-off, either based on the Vandamme model or on a fixed angle. I defined this cut-off algorithm as *dynamic* because, to provide a more reliable filtering, it also includes the correction for inclination shallowing of directions (if present) within the reiterative process (unless specified otherwise in the command line) (Fig. 2). Including the inclination shallowing correction within the reiterative process makes the cut-off itself less “strict” and more realistic. This is represented visually in Fig. 3. Two fixed cut-off angles of 40° and 45° are applied to the VGPs of the *Ardo_PmagDiR* record before (Fig. 3A) and after (Fig. 3B) correction for inclination flattening, as it would happen with the first re-iteration of the *PmagDiR::cut_DI* algorithm. The insets indicate the number of directions (VGPs) filtered by the two cut-off angles, and they are systematically lower after inclination flattening correction. At the end of the process, the *cut_DI* function returns the filtered directions in the input coordinates and not corrected for the inclination flattening. The algorithm is illustrated in the block model of Fig. 2A. Results of both the Vandamme and a fixed (40°) *dynamic* cut-off using the reference *Ardo_PmagDiR* paleomagnetic directions dataset are shown in Fig. 2B.

### Reversal test

**Figure 4:**
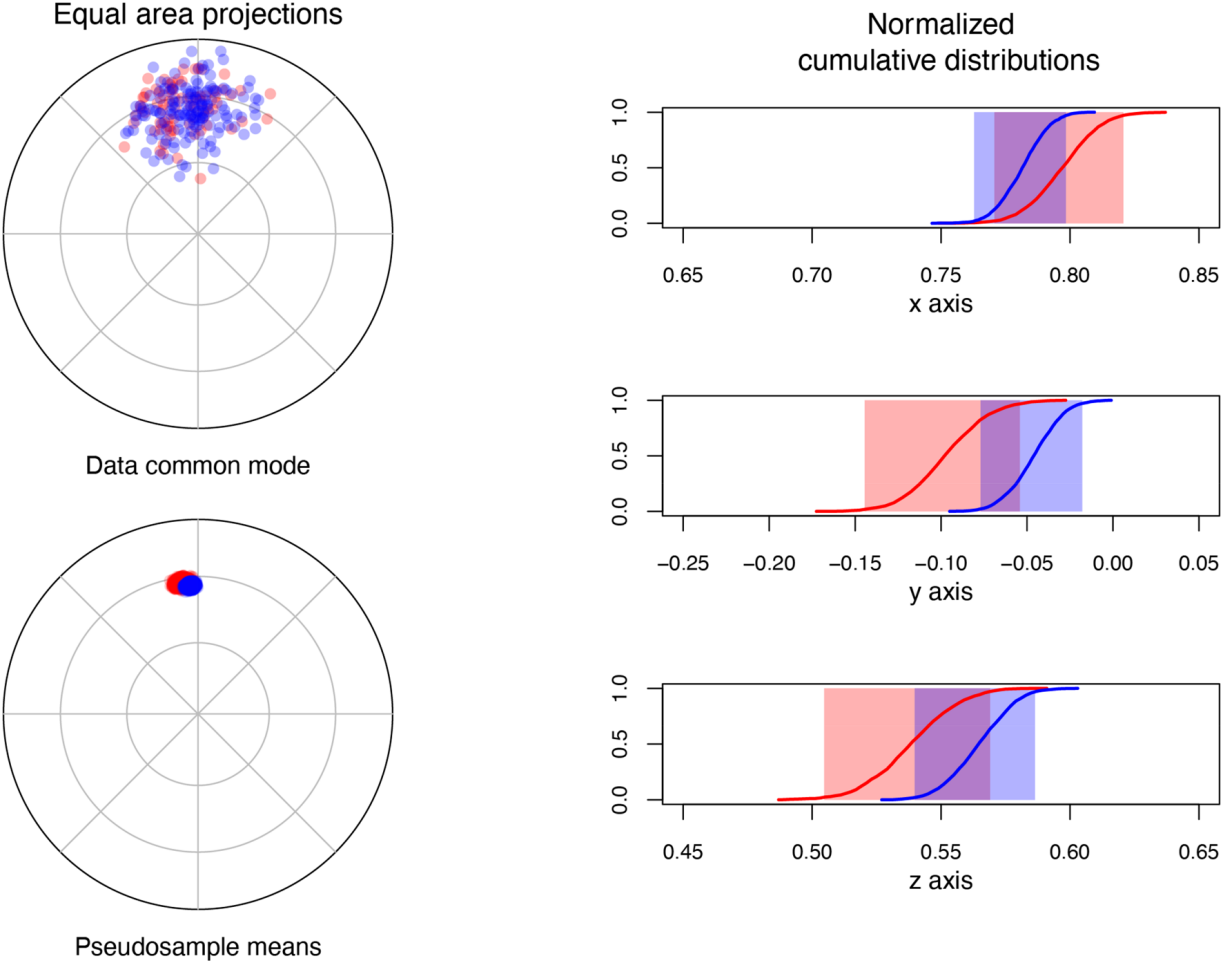
Bootstrap-based reversal test^30^ of the reference directions distribution Ardo_PmagDiR available within the PmagDiR package after application of the Vandamme^28^ cut-off. The figure shows the single vector file automatically exported after execution of the test. Confidence margins, calculated in this case by analyses of 2000 bootstrapped pseudosamples, are also exported as comma separated values file.

A set of paleomagnetic directions encompassing a time interval straddling one or more geomagnetic reversals is expected to be bimodal. Under the condition of a dipolar-dominated and axisymmetric geomagnetic field, the average directions of the two modes are expected to be antipodal^48,49^. The test for antipodality of paleomagnetic directions has been used extensively to assess the reliability of the paleomagnetic remanence since the pioneering years of the discipline^50–52^. Such tests are traditionally based on the assumption that directions are Fisher distributed, which implies a uniform distribution around the mean direction and the occurrence frequency of the directions decaying exponentially with the angular distance from the mean^2,36^. Even if this hypothesis can be tested^22,36^, the geomagnetic field geometry and the expected paleosecular variations should lead to paleomagnetic directions distributions that (with the exception of very high latitudes) have a certain degree of elongation^49^. A Fisher-based test is then theoretically not applicable on paleomagnetic distributions unless the Fisher-distribution hypothesis has not been tested before. To overcome this limitation, Tauxe et al. (1991)^30^ developed a bootstrap-statistic-based reversal test which I adopt in the *PmagDiR* package. A number of pseudosamples (*PmagDiR::revtest* default is 1000 but it can be defined by the user) are drawn from the paleomagnetic directions of the two modes, both plotted on a common down-pointing polarity. The Cartesian coordinates of the bootstrapped means are plotted in three separate plots, one for each axis, with the occurrence frequency shown as normalized cumulative distributions. The 95% confidence along each axis is bound by the 0.025 and the 0.975 element (for example, in the case of 1000 bootstrapped pseudosamples, by the 25^th^ and the 975^th^ ranked samples). The hypothesis of antipodality can be rejected if the confidence intervals of the two modes along any of the axes do not overlap. The *PmagDiR::revtest* can be easily performed after the application of directions cut-off, and the resulting graphic, which includes the direction distribution plotted on a common mode and the bootstrapped pseudosamples means, can be readily exported in a single vector file (Fig. 4).

### Shape of paleomagnetic distributions

In a seminal paper, Tauxe and Kent (2004)^37^ proposed a paleosecular variations model (TK03.GAD) that predicts the shape (elongation) of a paleomagnetic directions distribution as a function of paleolatitude (inclination). This assumption is at the base of the detection and correction of paleomagnetic inclination flattening (typical of sedimentary rocks) applied in many studies^10,34,53–57^. The TK03.GAD paleosecular variations model predicts that paleomagnetic directions distributions possess a degree of inclination-dependent elongation (E), which reaches a maximum at equatorial latitudes. This elongation is oriented parallel to the mean direction (i.e., parallel to the Earth’s meridians in case of an unrotated sampling site). The elongation of a distribution, as well as its declination with respect to the mean direction (Edec), can change under the influence of different natural “Earth’s filters” such as sedimentary compaction or tectonic strain^31,49^. Therefore, E and Edec can be used to assess the reliability of a paleomagnetic directions distribution.

**Figure 5:**
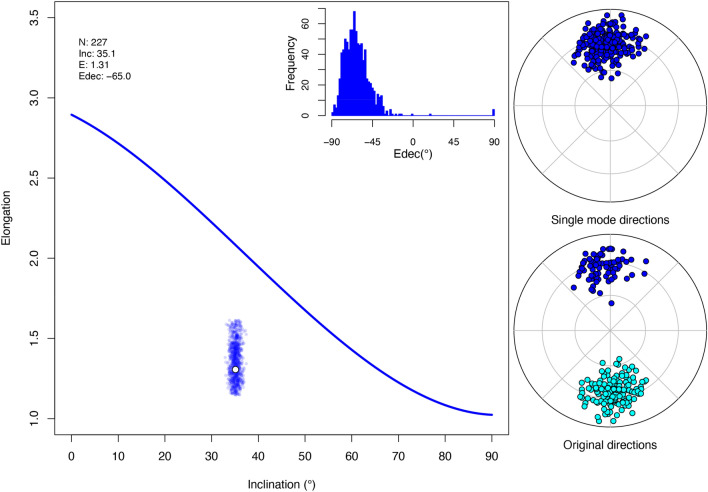
Result of the distribution shape analysis and the 95% confidence boundaries (calculated from 1000 bootstrapped pseudosamples) of the reference Ardo_PmagDiR dataset (after dynamic Vandamme cut-off). *N* number of directions, *Inc.*  average inclination, *E* elongation, *Edec* declination of elongation with respect to the average declination of the distribution. The analysis is shown with the equal area projection of the dataset, in the original form (automatically plotted only in the case of the bimodal distribution) and plotted on a single (down-pointing) mode.

The *PmagDiR* package helps with this process by plotting the elongation-inclination (E-I) pair of a paleomagnetic directions distribution, Edec, as well as the results obtained from the analysis of a defined number (default is 1000) of bootstrapped pseudosamples. Figure 5 shows this analysis performed on the *Ardo_PmagDiR* reference data (after directions cut-off as described above). The E-I pair falls well below the values predicted by the TK03.GAD model and Edec is significantly apart from the expected (~ 0°) declination. This is typical of a distribution affected by sedimentary inclination flattening^58^.

## Correcting flawed distributions

### Paleomagnetic inclination flattening

Inclination flattening of paleomagnetic directions carried by sediments and sedimentary rocks is a long-known problem. The observed (flattened) paleomagnetic inclination (I_f_) and the inclination of the inducing filed (I_o_) are related by the tangent function^59^:

1 $${\text{tan}}{I}_{f}=f\cdot {\text{tan}}{I}_{o}$$

where *f* is the inclination flattening factor ranging from 0 (completely flattened directions) to 1 (absence of flattening). Typically, *f* varies from 0.4 to 0.9 depending on the degree of compaction, presence of clay minerals and the magnetization carrier^56^. Common approaches for detecting and correcting for inclination shallowing are based on the magnetic fabrics measured in rocks, in the form of either anisotropy of magnetic susceptibility (AMS) or anisotropy of anhysteretic and isothermal remanent magnetization (AARM and AIRM, respectively)^57,60–63^.

Starting from the relationship between inclination and elongation expected by the TK03.GAD model, Tauxe and Kent (2004)^37^ developed an alternative strategy where all directions of a distribution are “unflattened” using Eq. (1) until the E-I pair assumes the values expected by the model. Despite some limitations (i.e. reliable results require a minimum of 100–150 high-quality paleomagnetic directions^46^) this method has been successfully applied on paleomagnetic records of different natures and age^10,34,53–58,64^.

**Figure 6:**
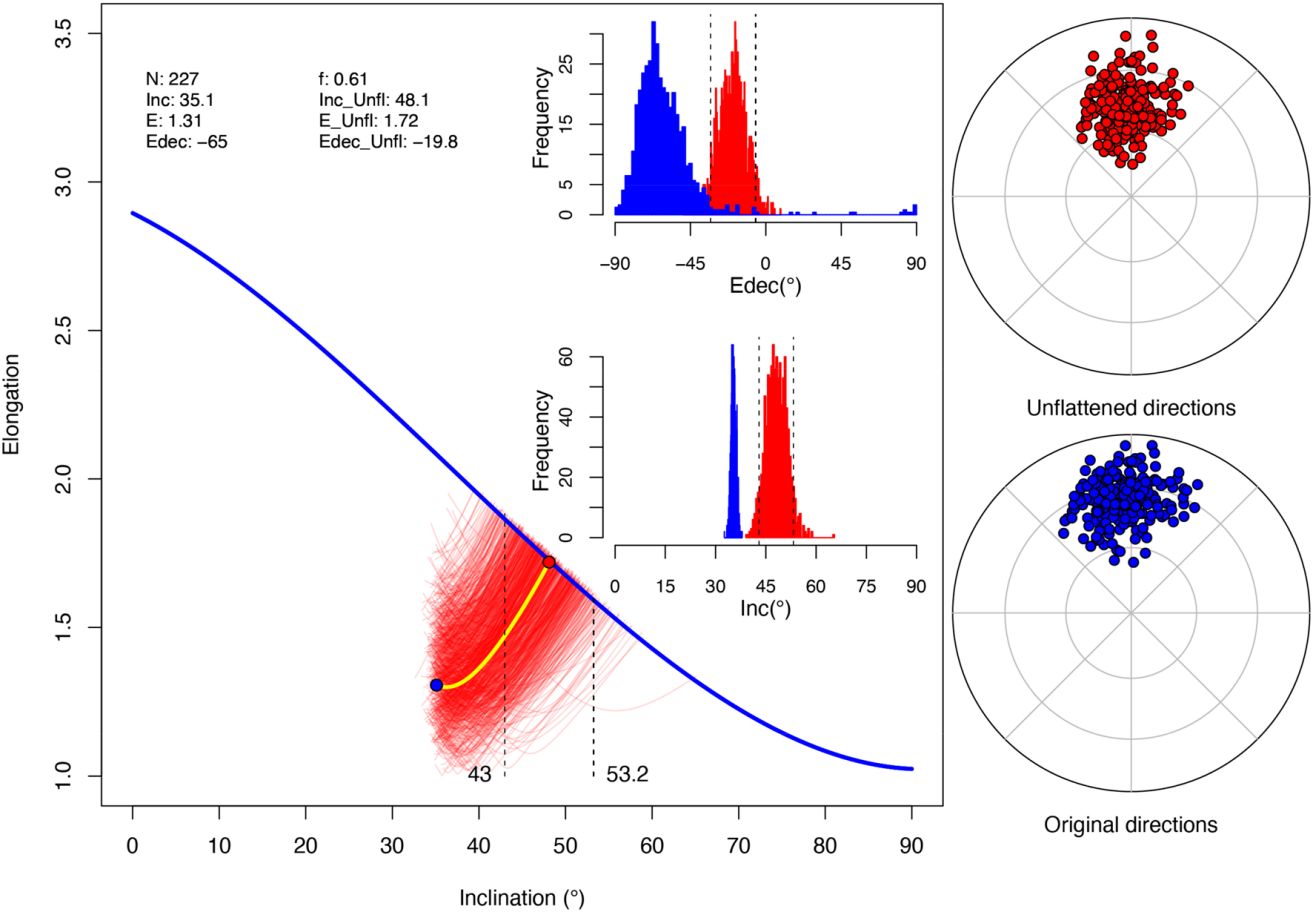
Paleomagnetic inclination flattening correction of the Ardo_PmagDiR directions distribution after applying the Vandamme dynamic cut-off. Numerical results are listed at the top left and exported as a text file with 95% confidence boundaries: *N* number of directions, *Inc and Inc_Unfl* original and unflattened inclinations, *E and E_Unfl* original and unflattened (target) elongation, *Edec and Edec_Unfl* original and unflattened declination of elongation with respect to the mean direction. The thick blue line is the TK03.GAD elongation vs inclination (E–I) function. The E–I pattern during unflattening of real data is shown by the thick yellow line, while the thin red curves are E–I pattern curves of 1000 (default) bootstrapped pseudosamples. Dashed lines are the lower and upper unflattened inclination confidence boundaries (indicated numerically). Results are shown with the frequency histogram of Inc and Edec of the bootstrapped pseudosamples, as well as the equal area diagrams of the original and unflattened directions (also exported as text file).

The *PmagDiR* package can perform the E-I test and, analogous to the original method implemented within the *PmagPy* package^22^, the confidence boundaries (95%) are determined through bootstrap statistic, with an arbitrary number of pseudosamples (default is 1000). Results are presented in a vector figure (automatically exported unless specified), together with text files including the bootstrap statistic and the “unflattened” directions (Fig. 6).

### Unstrain paleomagnetic directions

Many rocks are affected by tectonic strain that, especially if weak, is not always detected by simple field observations and can deviate paleomagnetic directions from the original orientation^33,65,66^. The finite strain state of a rock can be qualitatively evaluated by using, among other rock-fabric measurements, AMS. By studying the AMS of sedimentary rocks, Parés et al. (1999)^67^ found that, during incipient deformation, the susceptibility fabric evolves from oblate (k_1_ ≅ k_2_ > k_3_, where k_1_, k_2_, and k_3_ are respectively the major, intermediate, and minor axes of the AMS tensor) with vertical k_3_ (typical AMS of undeformed compacted sediments) to a triaxial k_1_ > k_2_ > k_3_ form with the minimum axis parallel to the shortening direction (strong cleavage state).

To restore paleomagnetic directions of sedimentary rocks, attempts were done in the past by using an “unstrain” strategy^32,33,68^. Paleomagnetic directions are considered as behaving like a passive line within the rock matrix, even though in some cases this approach is an oversimplification^66,69^. In this method the quality and reliability of the “unstrained” dataset is assessed by using basic Fisher^27^ statistical criteria, whereby increasing of the precision parameter (k, for details see the computational background in the Supplementary File available online) reflects improved clustering of the directions. However, the remanence magnetization of rocks reflects the more complex behavior of the geomagnetic field, and using just the standard spherical statistics to evaluate the reliability of paleomagnetic datasets adds further uncertainties. Similar to the paleomagnetic inclination flattening, pervasive strain leads to E-I couples of values that are “unrealistic”, departing from the values expected from the TK03.GAD field model.

*PmagDiR* offers the possibility not only to monitor the shape of the paleomagnetic directions distribution (Figs. 4 and 5), but also to gradually “unstrain” the directions while monitoring the E-I pair as well as the elongation declination Edec. Due to the non-unique solution of the unstrain problem, a fundamental pre-requisite is to know the degree and, most importantly, type and orientation of the rock-strain fabric. If such datum is available, in the form of, for example, AMS, *PmagDiR* can calculate the eigenvectors of the reversed strain matrix (function *AMS_inv*) to be used in the unstrain process. A target degree of foliation (Fol) and lineation (Lin; see Supplementary File for computational details) are set before starting the process. As the E-I pair before natural strain is unknown (it can fall either below or above the reference curve, see example Fig. 5), it is not guaranteed that the E-I curve during “unstrain” will cross the value expected by the TK03.GAD model (condition that, for example, stops the unflattening protocol for the final result; Fig. 6). For this reason, the unstrain process continues until the target Fol and Lin are reached. After monitoring the final behavior of the dataset, one of the following three conditions can be set to break the process of the most likely pre-strain distribution:

- *TK03.GAD crossing point*: analogous to the unflattening function, the process stops when the E-I pairs curves cross the value predicted by the paleosecular variations reference TK03.GAD model.
- *Edec minimalization*: Edec should approach zero in case of undisturbed rocks. The unstrain process can be stopped at the most likely E-I pair when the Edec angle is minimalized.
- *Edec maximalization*: on the contrary, directions distribution from sedimentary rocks, especially in the case of clay-rich and carbonate-poor rocks^54^, or magnetic remanence carriers by detrital hematite^58,70^, are likely to possess an elongation that is perpendicular to the mean direction (Edec approaching 90°), also due to the compaction flattening (see Fig. 5). The unstrain process can be stopped at the most likely E-I pair when the Edec angle is maximized.

Once the best process for the real dataset has been selected, the uncertainty (95% by default) can be estimated by repeating the analysis on an arbitrary number of bootstrapped pseudosamples.

### Unstrain real data

**Figure 7:**
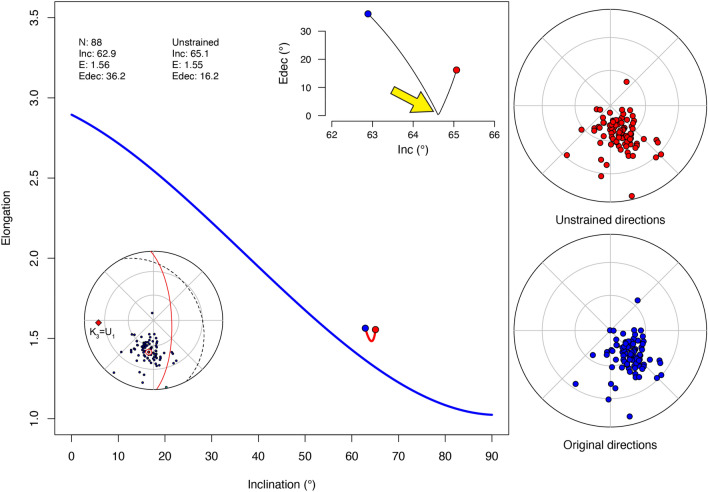
Elongation–inclination (E–I) and declination of elongation (Edec) paths inset in the main panel of the unstrained reference km_PmagDiR paleomagnetic directions. Unstrain is performed by the function PmagDiR::unstr_DI using a progressive unstrain series of matrices where eigenvectors are determined from the inverse anisotropy of magnetic susceptibility (AMS) tensor as described in the text (computational details are explained in the Supplementary File). The equal area projection inset (added manually to the original exported figure) shows the km_PmagDiR directions before correction of bedding tilt, the bedding plane (dotted line), and the orientation of the AMS tensor, where k_3_ (minimum AMS axis; intermediate and maximum axes are statistically equal and lie on the red circle) coincides with u_1_ (the maximum axis of the applied unstrain tensor). Blue and red dots in the main diagram are the initial and final E–I pairs, respectively. The yellow thick arrow (added to the original exported figure) marks the kink of the Edec path where Edec is minimalized (i.e. target unstrain degree shown in Fig. 8).

**Figure 8:**
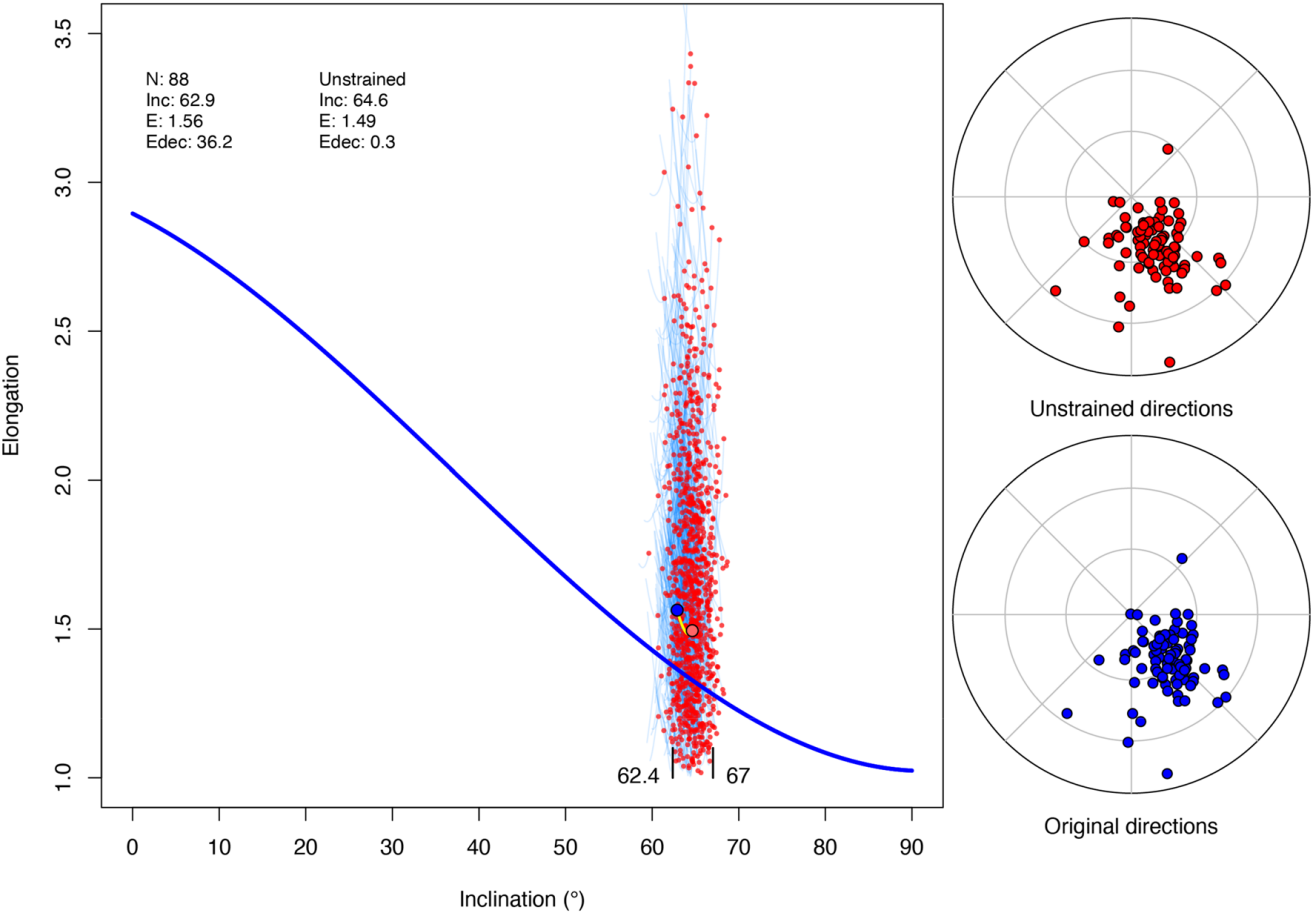
The main panel shows the unstrain path (short yellow line), initial (blue dot) and final (pale-red dot) elongation-inclination (E–I) pairs of the km_PmagDiR reference distribution after stopping the process when the declination of elongation (Edec) is minimalized (Fig. 7). The same analysis is repeated (function PmagDiR::unstr_boot) on 1000 bootstrapped pseudosamples drawn from the km_PmagDiR distribution. The cyan lines are the E–I pairs path ending with the red dot of the bootstrapped pseudosamples. The 95% confidence boundaries of the final inclination are indicated in the bottom of the main panel. The equal area plots are the km_PmagDiR directions before (blue) and after (red) unstrain.

To demonstrate the functionality of the unstrain process using *PmadDirs*, the paleomagnetic data from the Eocene sedimentary rock section exposed near Koumac (northern New Caledonia^31,39^) is shown (the procedure and all commands are explained step by step in the Workflow Example available in the Supplementary File). A representative set of specimens from the section were measured for AMS to obtain a statistically oblate fabric, which, having the minimum k_3_ axis not perpendicular with the bedding plane (inset in Fig. 7), is very likely to indicate tectonic strain. Other field geology observations support this hypothesis^39^. First, starting from the AMS tensor I calculated a matrix of the inverse eigenvectors by using the *PmagDiR::AMS_inv* function. Being the AMS oblate (i.e., k_1_ ≅ k_2_ > k_3_), I applied the unstrain “U” matrix in the form of u_1_ > u_2_ = u_3_ with progressively higher degrees of lineation (u_1_/u_2_) ranging from 1 to 1.5 (a value that significantly overcomes the AMS foliation degrees by about 1.01), keeping u_1_ parallel to the k_3_ AMS axis. As the bedding is also a physical entity affected by the tectonic strain^66^ the unstrain process is applied also the bedding plane. As shown in Fig. 7, while the E-I pairs vary minimally through the process, not approaching the reference curve, the Edec parameter reaches a minimum (between 64 and 65° of inclination, marked by the yellow arrow in Fig. 7). This minimum (i.e., Edec parallel with the mean declination) represents the most likely solution. The process can be performed again and stopped when Edec is minimalized, repeating the analysis on 1000 bootstrapped pseudosamples (Fig. 8). From this analysis results show that the unstrain process does not significantly affect the paleomagnetic inclination, as the initial inclination is included in the 95% confidence boundaries of the unstrained one. A similar approach was already adopted on the same dataset^31^, but *PmagDiR* now offers the possibility to perform the analysis with more precise constraints on both the real data and the bootstrapped pseudosamples.

It is important to highlight that, although implemented for *PmagDiR*, this unstrain strategy is, to date, not extensively tested with real data. Future research will demonstrate whether tectonically biased paleomagnetic directions can successfully be restored.

## Plotting virtual geomagnetic poles

**Figure 9:**
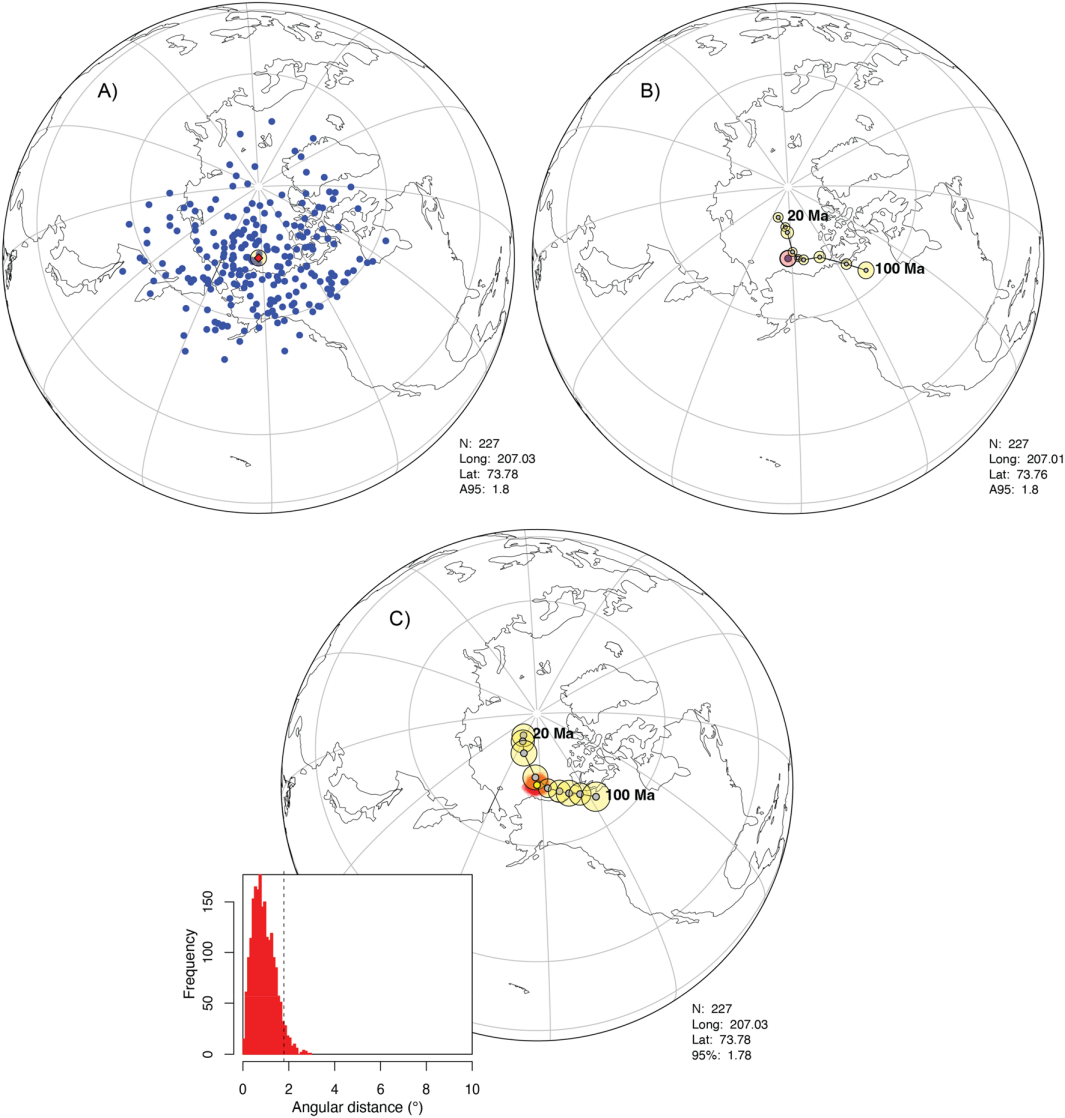
Spherical orthographic projections. (**A**) Virtual geomagnetic poles (VGPs) determined from the Ardo_PmagDiR reference paleomagnetic directions set after application of the dynamic Vandamme cut-off and correction for inclination flattening. The mean paleomagnetic pole is also indicated and automatically described in the exported figure (bottom-right). (**B**) The pole can be plotted together with the reference global apparent polar wander path of Vaes et al. (2023, V23_GAPWP^15^), in this case plotted from 20 to 100 Ma in the South African reference frame. (**C**) As in (**B**), but the 95% confidence angle is determined by analysis of 2000 bootstrapped VGPs pseudosamples, shown in the inset, and compared with the GAPWP of Torsvik et al. (2012, T12_GAPWP^16^).

A given set of paleomagnetic directions, either before or after filtering and correction, is normally converted into the spherical domain by calculating the VGP associated to each direction. *PmagDiR* converts directions into VGPs with different exporting formats including (1) bimodal VGPs with no manipulation, (2) VGPs converted into a single mode (used for paleomagnetic pole calculations), (3) VGPs with average coordinates centered on the spin axis (used for magnetic polarity stratigraphy). The average longitude and latitude of the VGPs cloud (paleomagnetic pole) is returned with the standard 95% Fisher^27^ cone of confidence. Data can be plotted with the global present-day coastlines for clarity (Fig. 9A). The paleomagnetic pole can be compared with two reference global APWPs (GAPWPs): the recently published GAPWP of Vaes et al. (2023^15^; *V23_GAPWP*, *PmagDiR* default) or the one of Torsvik et al. (2012^16^; *T12_GAPWP*), both included within *PmagDiR* as usable dataset*.* Through interactions with the RStudio console, the user can select the GAPWP age interval and one of the reference frames (Fig. 9B). An empirical approach for calculating the confidence angle of the paleomagnetic pole can be adopted using the function *PmagDiR::VGP_boot*, which estimates the 95% confidence by averaging a number (default is 1000) of bootstrapped VGPs pseudosamples. The angular distance of the pseudosamples mean directions are plotted as a frequency function within the exported figure (Fig. 9C) and a transect of the reference GAPWP can be plotted for comparison. This visual comparison represents the basis for future development of algorithms allowing quantitative comparison of between VGPs distributions and reference poles^13,71^.

## Other useful functionalities

A number of scripts within the current version *PmagDiR* package are used by the main processes described above, however they can also be useful tools as *standalone* functions for different calculations. These include:

- *bed_DI*: rotates declination-inclination pairs from geographic to tilt-corrected coordinates.
- *common_DI*: rotates all directions toward a common mode, either down- or up-pointing.
- *DI_from_VGP*: calculates paleomagnetic directions from VGPs and site coordinates.
- *flat_DI*: applies to the directions an arbitrary “f” flattening factor to obtain synthetically “flattened” paleomagnetic directions.
- *flip_DI*: “flips” directions to the antipodal.
- *Geo_point*: plots points (with circular area around if requested, like paleomagnetic poles) on a global geographic map.
- *PCA_DI*: returns mean declination, inclination and maximum angular deviation of demagnetization vector end-points by using principal component analysis^72^.
- *plot_a95:* a graphic function that plots mean directions and associated Fisher^27^ confidence on an equal area projection, with several options.
- *plot_PA95:* a graphic function that plots paleomagnetic poles and associated Fisher^27^ confidence on a spherical orthographic projection with several options, including present day coastlines and reference apparent polar wander paths.
- *Plot_plane*: plots planes and associated poles on an equal area diagram.
- *strain_DI*: simulates the effect of strain on paleomagnetic directions by applying a user-defined deformation (strain) matrix.
- *unflat_DI*: corrects paleomagnetic directions by applying a user-defined “f” flattening factor.

Details about each script of *PmagDiR* can be explored by typing “?*Name_of_script*” in the command line of the R-Studio console. A useful functionality of RStudio is that scripts can be nested, avoiding multiple lines of commands. For example, the nested command:

- plot_DI(common_DI(bed_DI(Ardo_Geo_PmagDiR), down = F))

Returns an equal area diagram of the *Ardo_PmagDiR* example dataset, corrected for bedding tilt and plotted on a common up-pointing (down = F, where F stands for FALSE) polarity.

## Conclusions

Paleomagnetic analysis of data and their graphical representation are fundamental for presenting results, and their reliability, to the scientific community. This first version of the *PmagDiR* package is designed to help the users perform reliability tests on paleomagnetic directions, as well as present their data in a clear and graphically appealing way, with minimal further graphical editing. R is an open-source programming language designed for statistical computing and graphics (https://www.r-project.org/) widely used in different disciplines of Earth science. *PmagDiR* is specifically designed to be optimally used with RStudio Desktop (https://posit.co/download/rstudio-desktop/). The workflow example available in the Supplementary File provided can help the user to install the package and work with the main commands.

### Supplementary Information

Supplementary Information 1.

Supplementary Information 2.

## Data Availability

The PmagDiR package in its current (and future) version is freely available on GitHub (https://github.com/edoardo-paleomag/PmagDiR).
