# Supplementary material for: Assessing the reliability of paleomagnetic datasets using the R package PmagDiR
Source: Sci Rep. 2024 Jan 18;14:1666. doi: 10.1038/s41598-024-52001-x (PMC10796433; doi:10.1038/s41598-024-52001-x)
Supplement: Supplementary file 1 — Supplementary Information 1. [file 41598_2024_52001_MOESM1_ESM.docx]

**Assessing the reliability of paleomagnetic datasets using the R package PmagDiR**

Edoardo Dallanave^1^

^1^Faculty of Geosciences, University of Bremen, Bremen, Germany ([edoardo@uni-bremen.de](mailto:edoardo@uni-bremen.de))

**Computational background**

**Directions mode determination**

Directions, given as declination (*D*) and inclination (*I*) pairs, are first converted in their Cartesian coordinates (*x_1_*, *x_2_*, *x_3_*) by using:

$x_{1i}=B\cos D_{i}\cos I_{i}$ (1)

$x_{2i}=B \sin D_{i}\cos I_{i}$ (2)

$x_{3i}=B \sin I_{i}$ (3)

where “*i*” ranges from 1 to N, with N= number of directions, and B is the length of the vector (considered as unit except after unstrain as described below). These data are used to calculate the orientation matrix^1^. Because the center of the distribution is assumed coinciding with the origin of coordinate system, the orientation matrix (T) is simplified as:

$T=\left[ \begin{matrix} \sum x_{1i}x_{1i} & \sum x_{1i}x_{2i} & \sum x_{1i}x_{3i} \\ \sum x_{2i}x_{1i} & \sum x_{2i}x_{2i} & \sum x_{2i}x_{3i} \\ \sum x_{3i}x_{1i} & \sum x_{3i}x_{2i} & \sum x_{3i}x_{3i} \end{matrix} \right]$ (4)

The eigenvector of maximum variance (*e_1_*) represents the average direction of the whole (one mode or bimodal) distribution. This is calculated by using the package R package *matlib* (<https://cran.r-project.org/web/packages/matlib/index.html>)*.* The Cartesian coordinates of *e_1_* (*e_11_*, *e_12_*, and *e_13_*) are converted back to spherical (*D_e1_*, *I_e1_*) by:

$D_{e1}= \tan^{-1} \frac{e_{12}}{e_{11}}$ (5)

$I_{e1}=\sin^{-1} \frac{e_{13}}{\sqrt{{e_{11}}^{2}+{e_{12}}^{2}+{e_{13}}^{2}}}$ (6)

The angular distance (θ_i_) of each direction from *e_1_* is calculated by using

$\theta_{i}=\cos^{-1} \left( \sin I_{e1}\sin I_{i}+\cos I_{ei}\cos I_{i}\cos\left( D_{ei}-D_{i} \right) \right)$ (7)

The angle *θ* is use as criterion to define the mode of each direction, whereby directions associated to *θ < 90°* belong to *mode 1* while directions with *θ ≥ 90°* belong to *mode 2.*

**Average paleomagnetic direction and confidence**

Considering each direction as a unit vector, the sum R is determined by:

$R=\sqrt{\left( \sum_{i} x_{1i} \right)^{2}+\left( \sum_{i} x_{2i} \right)^{2}+\left( \sum_{i} x_{3i} \right)^{2}}$ (8)

From this the average cartesian coordinates are:

$\bar{x}_{1}=\frac{1}{R}\left( \sum_{i} x_{1i} \right); \bar{x}_{2}=\frac{1}{R}\left( \sum_{i} x_{1i} \right); \bar{x}_{3}=\frac{1}{R}\left( \sum_{i} x_{1i} \right)$ (9)

Average declination ($\bar{D})$ and inclination ($\bar{I})$ are calculated by:

$\bar{D}= \tan^{-1} \frac{\bar{x}_{2}}{\bar{x}_{1}}$ (10)

$\bar{I}=\sin^{-1} \frac{\bar{x}_{3}}{\sqrt{{\bar{x}_{1}}^{2}+{\bar{x}_{2}}^{2}+{\bar{x}_{3}}^{2}}}$ (11)

Knowing the number of directions (N), the Fisher^2^ precision parameter (k) is estimated with:

$k=\frac{N-1}{N-R}$ (12)

The semi-angle of the 95% cone of confidence (α_95_) is calculated with:

$\alpha_{95}= \cos^{-1} \left\{ 1-\frac{N-R}{R}\left[ \left( \frac{1}{0.05} \right)^{\frac{1}{N-1}}-1 \right] \right\}$ (13)

Analogously, the same procedure can be used to average the position of the virtual geomagnetic poles (VGPs) and estimate the 95% angle of confidence, referred to as A_95_. The semi axes of the confidence ellipse around the average direction, derived by the A_95_ following the rationale described in the main text^3^, are calculated with:

$\Delta D=\sin^{-1} \left( \frac{\sin A_{95}}{\cos\lambda} \right)$ (14)

$\Delta I=\frac{2A_{95}}{\left( 1+3\sin^{2} \lambda\right)}$ (15)

Where ΔD and ΔI are the confidence semi-angles of the declination and inclination (respectively), while λ is the paleolatitude calculated from the average inclination using the dipole formula:

$\lambda=\tan^{-1} \left( \frac{\tan\bar{I}}{2} \right)$ (16)

**Equal area diagrams**

Declination and inclination (*D*, *I*) are converted into the two dimensions cartesian (*x* and *y*) Cartesian plane by using:

$x=\sqrt{2}\cdot\frac{\sin\left( 90-I \right)}{2}\sin D$ (17)

$y=\sqrt{2}\cdot\frac{\sin\left( 90-I \right)}{2}\cos D$ (18)

**Conversion of directions into virtual geomagnetic poles**

For converting paleomagnetic directions in virtual geomagnetic poles (VGP) I adopted the trigonometrical calculation described by Butler^4^, whereby:

*D*= paleomagnetic direction declination

*I*= paleomagnetic direction inclination

λ_s_, φ_s_ = site latitude and longitude

λ_p_, φ_p_ = VGP latitude and longitude

*p* = great circle distance from site to VGP

*β* = longitudinal difference between site and VGP

After defining these parameters, the VGP coordinates are calculated:

$p=\tan^{-1} \left( \frac{2}{\tan^{-1} I} \right)$ (19)

$\lambda_{p}=\sin^{-1} \left( \sin\lambda_{s}\cos p+\cos\lambda_{s}\sin p\cos D \right)$ (20)

$\beta=\sin^{-1} \left( \frac{\sin p\sin D}{\cos\lambda_{p}} \right)$ (21)

If:

$\cos p\geq\sin\lambda_{s}\sin\lambda_{p}$ (22)

Then:

$\phi_{p}=\phi_{s}+\beta$ (23)

If:

$\cos p< \sin\lambda_{s}\sin\lambda_{p}$ (24)

Then:

$\phi_{p}=\phi_{s}+180-\beta$ (25)

**Spherical Orthogonal projection**

*PmagDiR* uses spherical orthogonal projections to plot VGPs and their average (paleomagnetic pole). If λ_o_ and φ_o_ are respectively the latitude and longitude of the projection center (point of view), while λ_I_ and φ_I_ the latitude and longitude of any point, the coordinates are converted into the two dimensions Cartesian (*x* and *y*) by using:

$x=\cos\lambda_{i}\sin\left( \phi_{i}-\phi_{o} \right)$ (26)

$y=\cos\lambda_{o}\sin\lambda_{i}-\sin\lambda_{o}\cos\lambda_{i}\cos\left( \phi_{i}-\phi_{o} \right)$ (27)

Whether a point plots “behind” the visible space of the spherical orthogonal projection is determined by:

$\cos c=\cos\lambda_{o}\sin\lambda_{i}+\cos\lambda_{o}\cos\lambda_{i}\cos\left( \phi_{i}-\phi_{o} \right)$ (28)

Whereby cos c < 0 indicates “hidden” point.

**Mapping tool**

The location of a geographic point or a paleomagnetic pole can be plotted on a Kavrayskiy VII projection, a compromise projection specifically developed for creating map with low distortion. Longitude (φ) and latitude (λ) are converted into the two dimensions Cartesian (*x* and *y*) by using:

$x=\frac{3\phi}{2}\sqrt{\frac{1}{3}-\left( \frac{\lambda}{\pi} \right)^{2}}$ (29)

$y=\lambda$ (30)

**Paleomagnetic directions cut-off**

Paleomagnetic directions cut-off of *PmagDiR* are based on the angular distance between the mean paleomagnetic pole and each VGP. The mean paleomagnetic pole is calculated applying standard spherical statistics^2^ (equations 8 to 11 using Longitude and Latitude instead of Declination (*D*) and Inclination (*I*)) on the VGPs flipped into a common mode. The angular distance between each VGP and the mean paleomagnetic pole is calculated applying equation (7). In case of the Vandamme^5^ cut-off, computational details are in the original publication.

**The TK03.GAD elongation equation**

The paleosecular variation model of Tauxe and Kent^6^ predict the Elongation (*E*) of a given paleomagnetic distribution as a function of the inclination (*I*). Elongation is calculated by the ratio of the intermediate over minimum eigenvalues (*E= v_2_/v_3_*) of the distribution matrix (4), while the expected E_tk03.GAD_ is determined by^7^:

$E_{tk03.GAD}= 2.895-\left( 1.466\cdot{10}^{-2}\cdot I \right)-\left( 3.525\cdot{10}^{-4}\cdot I^{2} \right)+\left( 3.16\cdot{10}^{-6}\cdot I^{3} \right)$ (31)

The declination of Elongation (*Edec*) used plotted and used by *PmagDiR* is the declination difference between the maximum (*e_1_*) and intermediate (*e_2_*) eigenvectors of the distribution matrix (4).

**Directions unstrain**

The first step of the unstrain process is calculating the eigenvectors matrix (M) of the anisotropy of magnetic susceptibility (AMS) inverse by using the function *PmagDiR::AMS__inv*. When calling the function *PmagDiR::unstr_DI*, the user must indicate the target lineation (L) and foliation (F) as explained in the main text. Knowing the relationships:

$L=\frac{s_{1}}{s_{2}}$ (32)

$F=\frac{s_{2}}{s_{3}}$ (33)

$P=F\cdot L$ (34)

$s_{1}{+s}_{2}{+s}_{3}=3$ (35)

where s_1_ ≥ s_2_ ≥ s_3_ are the three eigenvalues of the target unstrain matrix, they can be calculated by:

$s_{1}=3/\left( \frac{1}{L}+\frac{1}{P}+1 \right)$ (36)

$s_{2}={s_{1}}/L$ (37)

$s_{3}={s_{1}}/P$ (38)

Knowing that:

$S=\left[ \begin{matrix} s_{1} & 0 & 0 \\ 0 & s_{2} & 0 \\ 0 & 0 & s_{3} \end{matrix} \right]$ (39)

The unstrain matrix U is determined by:

$U=MSM^{-1}$ (40)

Converting each direction in Cartesian coordinates (x_1_, x_2_, and x_3_) with equations (1) to (3), the coordinate of each unstrained direction (d_1_, d_2_, and d_3_) are determined by:

$\left[ \begin{matrix} d_{1} \\ d_{2} \\ d_{3} \end{matrix} \right]=\left[ \begin{matrix} u_{11} & u_{12} & u_{13} \\ u_{21} & u_{22} & u_{23} \\ u_{31} & u_{32} & u_{33} \end{matrix} \right]\left[ \begin{matrix} x_{1} \\ x_{2} \\ x_{3} \end{matrix} \right]$ (41)

Where u_ij_ are the elements U.

The declination and inclination of the unstrained directions are calculated using equations (5) and (6).

**Bootstrapping**

The bootstrapped-based statistic used by *PmagDiR* is calculated by using the base function of R *base::sample.* Detailed information are obtained by typing “*?sample*” in the R-Studio console. The *base::sample* function allows resampling with repetition, which is based on the Walker’s alias method^8^.

**References**

1. Scheidegger, A. E. On the statistics of the orientation of bedding planes, grain axes, and similar sedimentological data. *U Geol. Surv. Prof. Pap.* **525**, 164–167 (1965).

2. Fisher, R. Dispersion on a sphere. *Proc. R. Soc. Lond.* **A217**, 295–305 (1953).

3. Deenen, M. H. L., Langereis, C. G., Van Hinsbergen, D. J. J. & Biggin, A. J. Geomagnetic secular variation and the statistics of palaeomagnetic directions: Statistics of palaeomagnetic directions. *Geophys. J. Int.* **186**, 509–520 (2011).

4. Butler, R. F. *Paleomagnetism: Magnetic Domains to Geologic Terranes*. (Blackwell Scientific Publication, 1992).

5. Vandamme, D. A new method to determine paleosecular variation. *Phys. Earth Planet. Inter.* **85**, 131–142 (1994).

6. Tauxe, L. & Kent, D. V. A simplified statistical model for the geomagnetic field and the detection of shallow bias in paleomagnetic inclinations: Was the ancient magnetic field dipolar? in *Timescales of the Paleomagnetic Field, Geophys. Monogr.* (eds. Channell, J. E. T., Kent, D. V., Lowrie, W. & Meert, J. G.) vol. 145 101–115 (American Geophysical Union, 2004).

7. Tauxe, L., Kodama, K. P. & Kent, D. V. Testing corrections for paleomagnetic inclination error in sedimentary rocks: A comparative approach. *Phys. Earth Planet. Inter.* **169**, 152–165 (2008).

8. Ripley, B. D. *Stochastic simulation*. (John Wiley & Sons, Inc., 1987).
