# Supplementary material for: Assessing the reliability of paleomagnetic datasets using the R package PmagDiR
Source: Sci Rep. 2024 Jan 18;14:1666. doi: 10.1038/s41598-024-52001-x (PMC10796433; doi:10.1038/s41598-024-52001-x)
Supplement: Supplementary file 2 — Supplementary Information 2. [file 41598_2024_52001_MOESM2_ESM.docx]

**Assessing the reliability of paleomagnetic datasets using the R package PmagDiR**

Edoardo Dallanave^1^

^1^Faculty of Geosciences, University of Bremen, Bremen, Germany ([edoardo@uni-bremen.de](mailto:edoardo@uni-bremen.de))

**Workflow example**

**Get started: download RStudio and the PmagDiR package from GitHub**

RStudio Desktop requires the installation of R. Updated links to both are found at the URL:

<https://posit.co/download/rstudio-desktop/>

*PmagDiR*(0.1.0) is currently archived on GitHub. To pull it from the repository through the RStudio console, one easy way is to use the *install_github* command of the *devtools* package. To do so, type these commands in the RStudio console pressing return after each command.

> install.packages("devtools“)

> library(devtools)

> install_github(“edoardo-paleomag/PmagDiR”)

To activate *PmagDiR* after successful download, type:

> library(PmagDiR)

> ?plot_Di

The last command should open a documentation page in the Help window of RStudio (Figure S1)


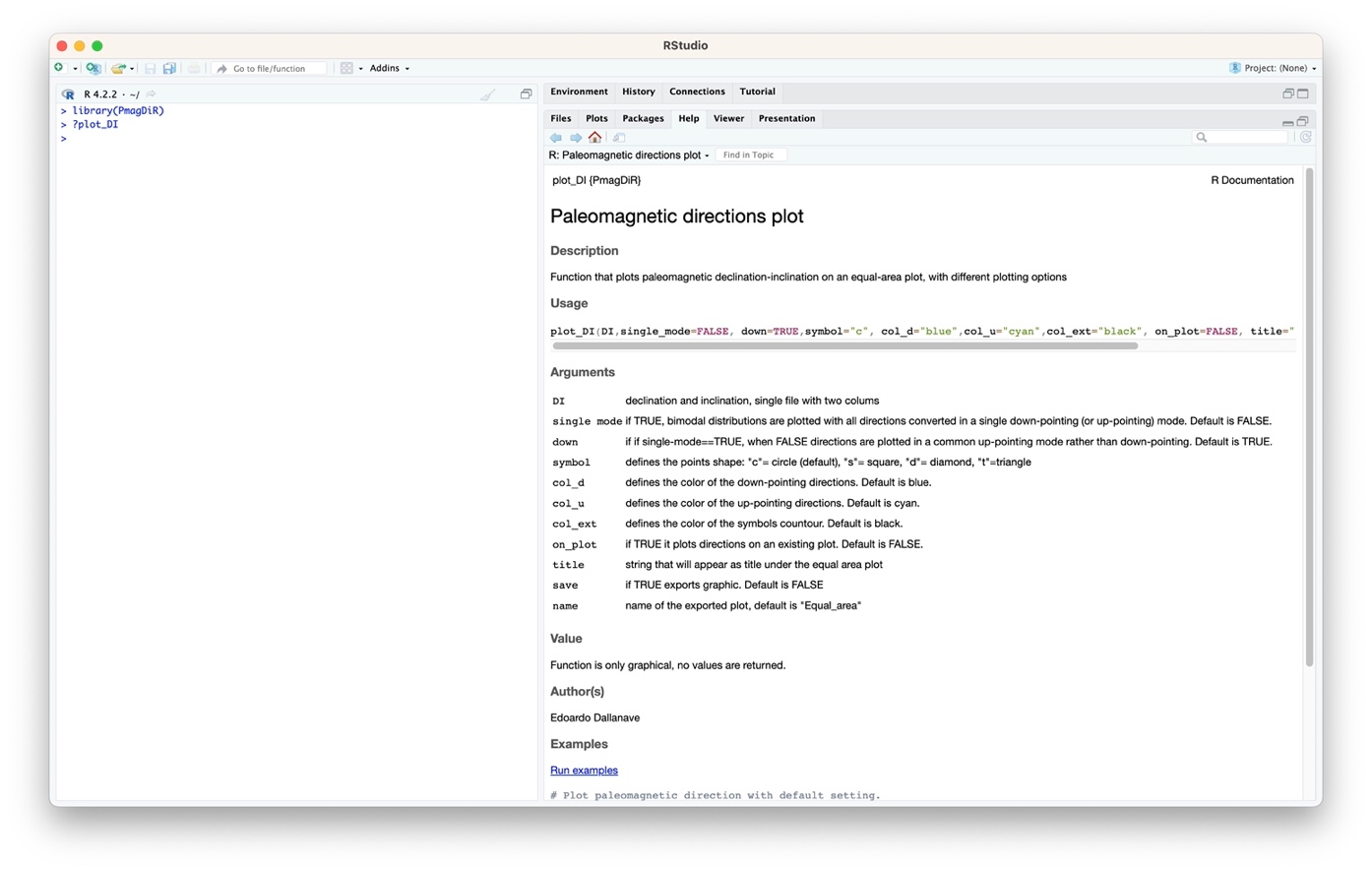


*Figure S1. Help window of RStudio. All the functionalities of a specific script (in this case PmagDiR::plot_DI) are described.*

By clicking on *Run examples* at the bottom of the help page, two equal area diagrams are displayed, both generated with the example datasets available within the package. The example data consist of paleomagnetic directions from the Italian Southern Alps^1^ (a dataset of paleomagnetic directions from Northern New Caledonia^2^ is also available). Information about the example dataset can be obtained as done for the single scripts, by typing:

> ?Ardo_PmagDiR

**Setting the working directory and importing files**

By using the *Files* window of RStudio, follow the path to the working directory of choice. Once in the directory, all files in the directory are listed. In order to have all automatically generated files and figures in this directory, it has to be set as *working directory* by selecting the option under the *More* menu (Figure S2).


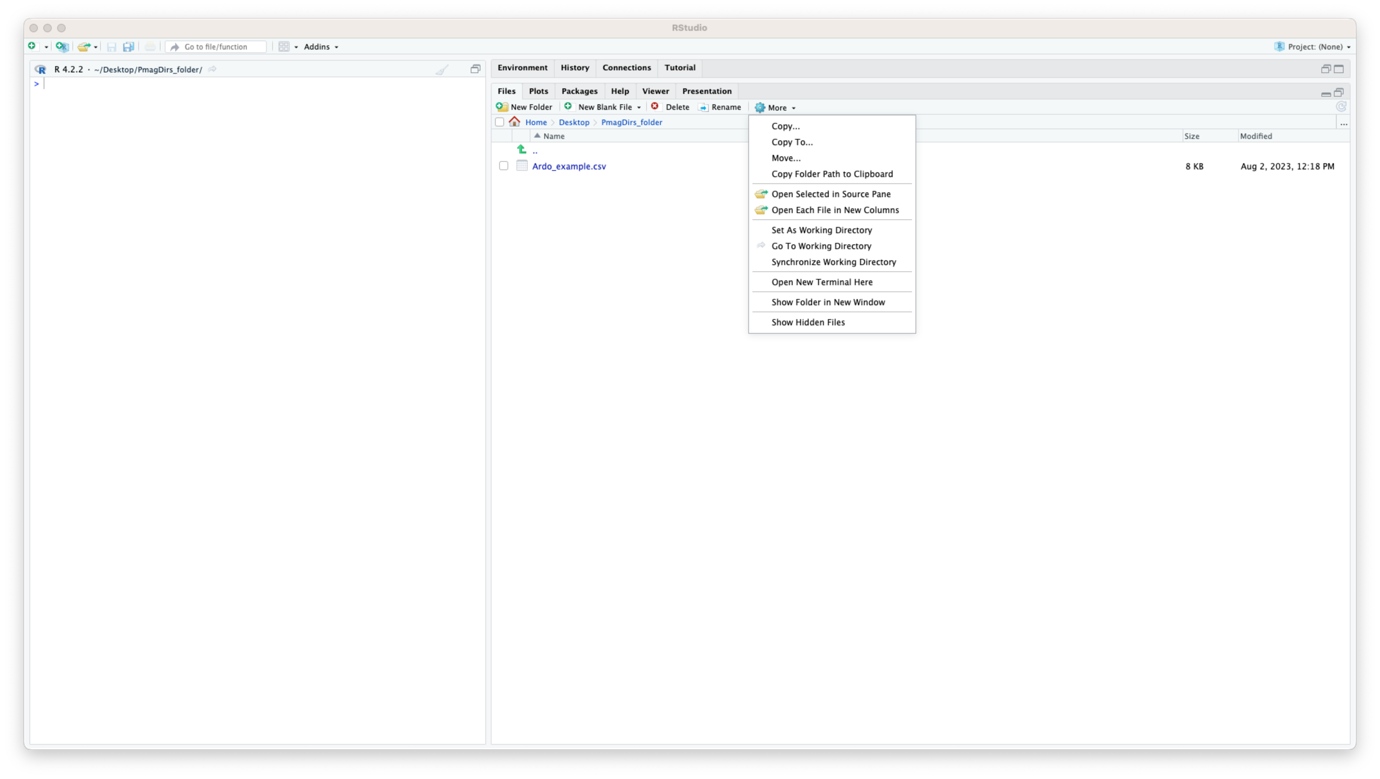


*Figure S2. Set the working directory.*

In the example of Figure S2, the directory contains a set of paleomagnetic directions (the same of *Ardo_PmagDiR*) that we wish to import in the RStudio Environment. An easy way to do so is to have them in comma separated value (.csv) format. The file can be imported (assigning a name, *ardo* in this case) from the command line by typing:

> ardo <– read.csv(“Ardo_example.csv”)

Alternatively, the command:

>ardo <– read.csv(file.choose())

allows interactive selection of the csv file by opening an exploring folder. A third alternative is clicking on the file within the RStudio *File* window and following the instructions. In case of problems using the symbol “<­–“ for assigning content to the RStudio Environment files, the symbol “=” is equivalent.

**Geographic location**

A simple geographic mapping tool is available within *PmagDiR* to locate sites (or paleomagnetic poles) on a map (Figure S3). The point is placed by interactive data entry in the RStudio console by typing:

> geo_point()

Some plotting options (e.g., grid spacing) can be defined within brackets and are detailed in the function documentation (by typing “*?geo_point”*).


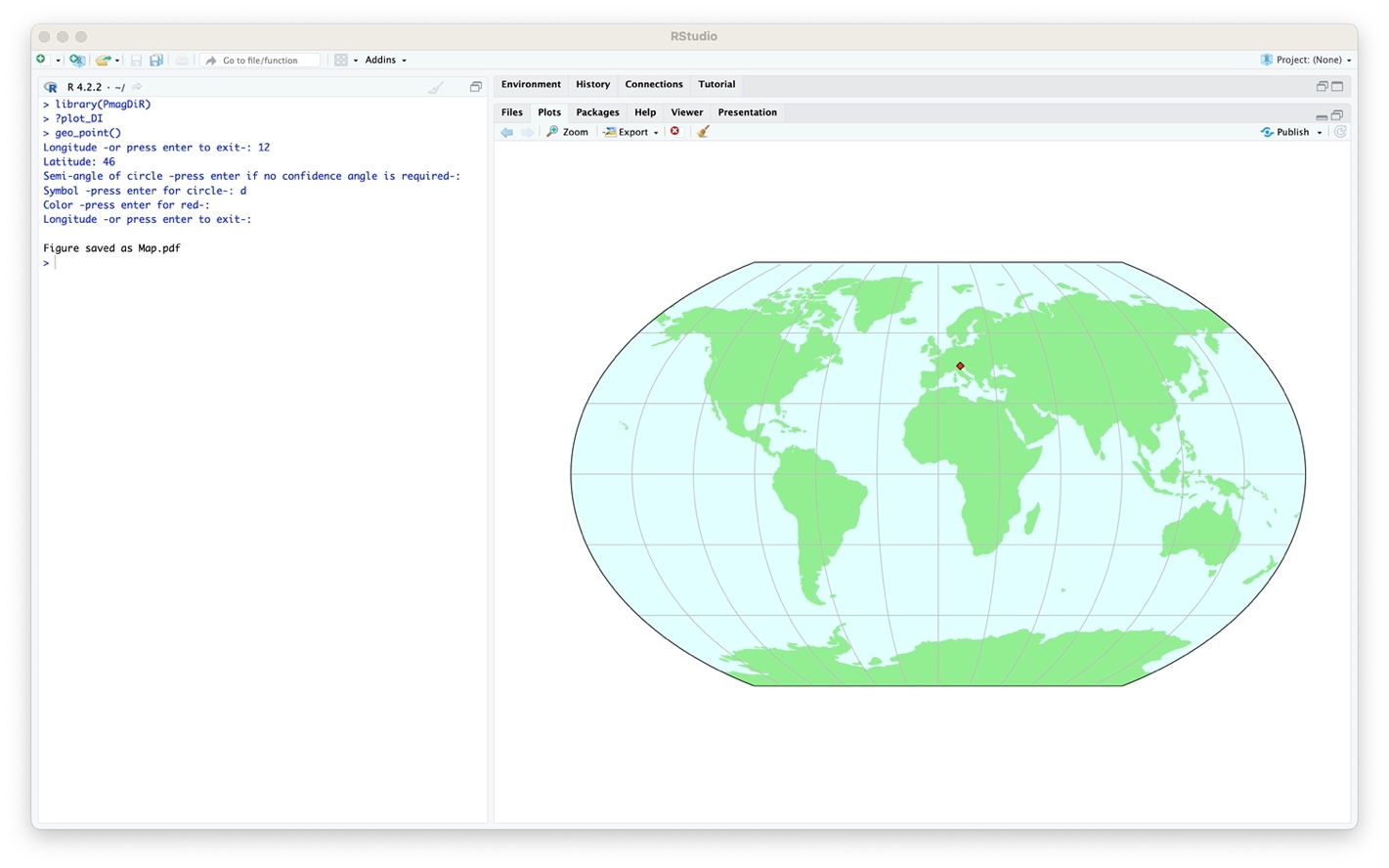


*Figure S3. Present day geographic location of the Ardo section.*

**Plotting, filtering, and correcting paleomagnetic directions**

Once the dataset is imported, we wish to visualize the data. This is done by typing:

> plot_DI(ardo)

This command will generate an equal area plot. All plotting options can be explored by typing ”*?plot_DI*”.

Fisher^3^ standard mean and confidence can be calculated and plotted on the same diagram (plotting on pre-existing diagram is default, see instructions by typing “*?fisher_plot*”) and result exported as *ardo_ALL.csv* file by typing the command:

> fisher_plot(ardo, export = T, name = "ardo_ALL", text=T)

This will generate the results shown in Figure S4 (files are saved in the working directory). The command *text=T* (equivalent of *text=TRUE*) will plot the main statistic result as text to the right bottom of the equal area diagram.


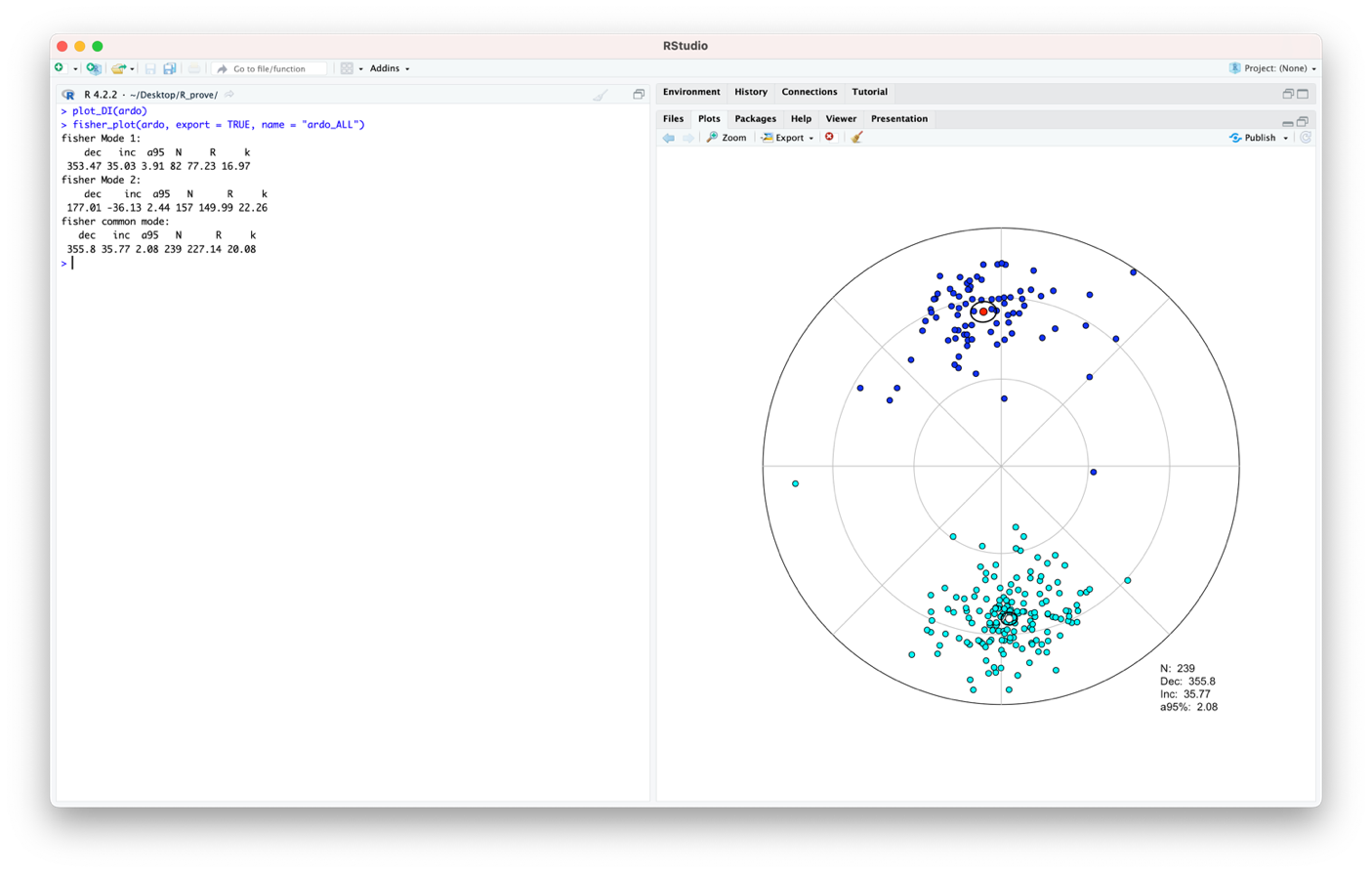


*Figure S4. Direction data plotting and Fisher*^3^ *statistic calculation.*

Some of the directions shown in Figure S3 are departing significantly from the two modes cluster. *PmagDiR* can apply a cut-off to exclude the potential outliers by using the *PmagDiR::cut_DI* function. Specifically, we wish to apply the cut-off proposed by Vandamme^4^ and store the filtered dataset in a new *ardoVD* file by typing the command:

> ardoVD <- cut_DI(ardo, lat = 46.04, long = 12.14)

where lat and long are latitude and longitude (respectively) of the Ardo section site^1^. Note that *PmagDiR::cut_DI* performs the Vandamme cut-off by default, and in order to apply a cut-off based on a fixed virtual geomagnetic pole (VGP) distance from the average paleomagnetic pole, the command “*VD=F*” (where F means FALSE) must be typed. The number of reiterations performed to filter the data is shown in the console. When applying this function, the new (*ardoVD* in the example case) file is not automatically exported in the working folder, unless specified in the *cut_DI* command line. This is done by typing *export = T* within the command. Text results that are present within the RStudio environment can be exported any time by using the command *write.csv* (type “*?write.csv”* for details).

The result of the cut-off can be evaluated visually by plotting both the original and the filtered dataset on the same equal area diagram. Different symbols and/or colors can be specified. For example, we can execute sequentially these commands:

> plot_DI(ardo, title = "Ardo cut-off")

> plot_DI(ardoVD, symbol = "s", col_d = "red", col_u = "pink", on_plot = T)

The first command produced the equal area with the whole dataset, while the second command plots the filtered data on top of the first equal area (for this reason the title of the diagram, if required, must be typed within the first command). The second command will plot directions as squares, red when down-pointing and pink when up-pointing. Analogously to the plot generated in Figure S3, standard fisher statistic can be calculated, plotted, and exported into the working directory (Figure S5).

> fisher_plot(ardoVD, save = T, name = "Ardo_VD", text=T)


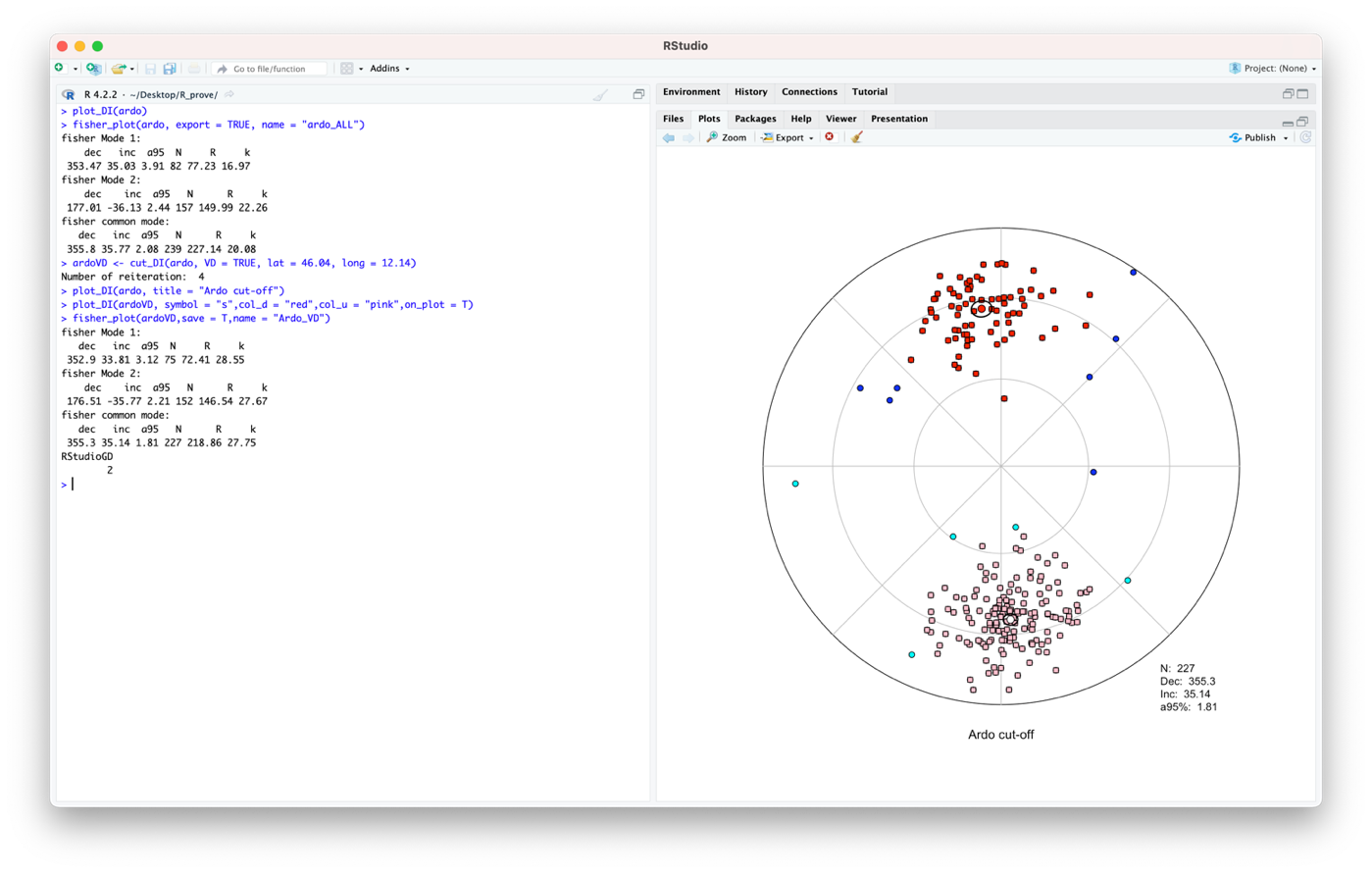


*Figure S5. Results of the Vandamme (1994) cut-off application and calculation of the Fisher (1953) statistic of the filtered dataset.*

We can now test the filtered dataset for antipodality. This can be performed by the function *PmagDiR::revtest* by typing:

> revtest(ardoVD,export = T, name = "Ardo_VD_revtest")

This will also automatically export the results and the figure in the working directory. When running the code, some packages will be automatically attached (potential conflicts warnings are deliberately left for users to check), then the calculation will be performed. The progressive number of bootstrapped datasets are also listed to monitor the process through (Figure S6).


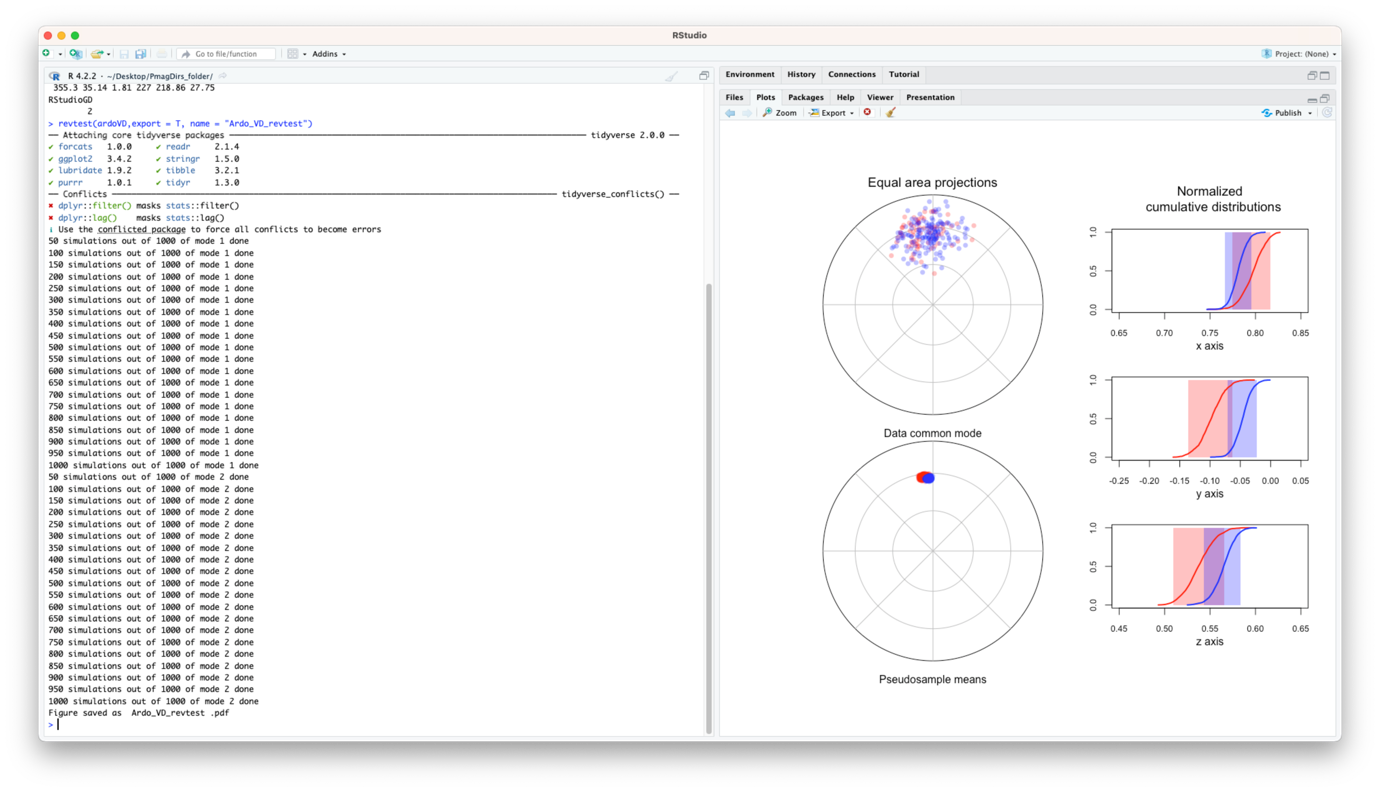


*Figure S6. Results of the bootstrap-based reversal test* (Tauxe et al., 1991)*. Overlapping cumulative distribution bands along the three axes indicate a positive reversal test.*

Once the antipodality has been attested, the reliability of the distribution shape, in terms of measured elongation-inclination pair versus the expected one, can be attested with the *PmagDiR::EI_boot* function. The bootstrapping process is relatively fast, so a number of simulations higher than the 1000 set by default can be selected (e.g., nb=2000):

> EI_boot(ardoVD,nb = 2000)

This gives the results of Figure S7.


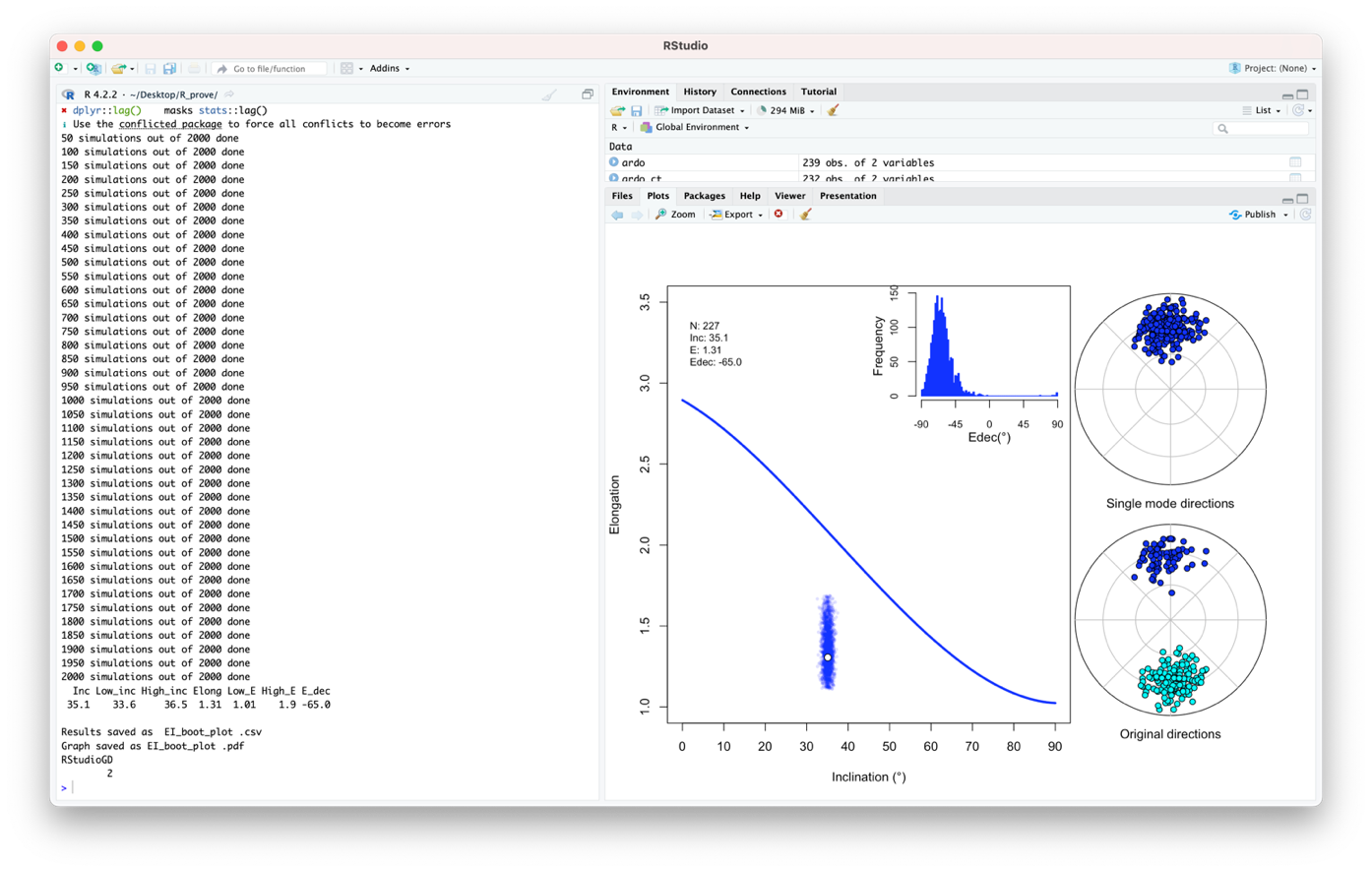


*Figure S7. Elongation-inclination pair of the ardoVD dataset compared with the values expected by the TK03.GAD paleosecular variation model*^5^ *(blue thick line). The cloud of blue points within the main diagram (Elongation-Inclination) envelops the 95% confidence area, while the declination of elongation of the 2000 bootstrapped pseudosamples is shown as frequency function. Depending on the proportions of the different windows, equal areas and main diagram may overlap. Nonetheless, the exported figure proportions are optimized to avoid this problem.*

Numerical data and confidence bounds are saved as .csv files, but from visual inspection of Figure S6 it results that the elongation of the distribution is significantly lower than the value expected from the TK03.GAD paleosecular variation model. Furthermore, the elongation declination (Edec) departs significantly from the expected ~0°. These data indicate “flattened” paleomagnetic directions, very likely the results of sedimentary compaction associated with hematite as magnetic remanence carrier^1^. The shallow bias inclination can be corrected by using the method proposed by Tauxe and Kent (2004)^5^, which has been successfully applied to many sedimentary paleomagnetic records (see references in main text). In synthesis, all directions are gradually flattened applying gradually decreasing values of flattening factor (f) within the formula tan (I_o_) = f · tan (I_o_), where I_o_ and I_o_ are respectively the observed and the original unflattened inclination. This can be performed by using the *PmagDiR::ffind_boot* function:

> ardo_unfl <– ffind_boot(ardoVD,name = "Ard_VD_unflattened")

that gives the results plotted in Figure S8.


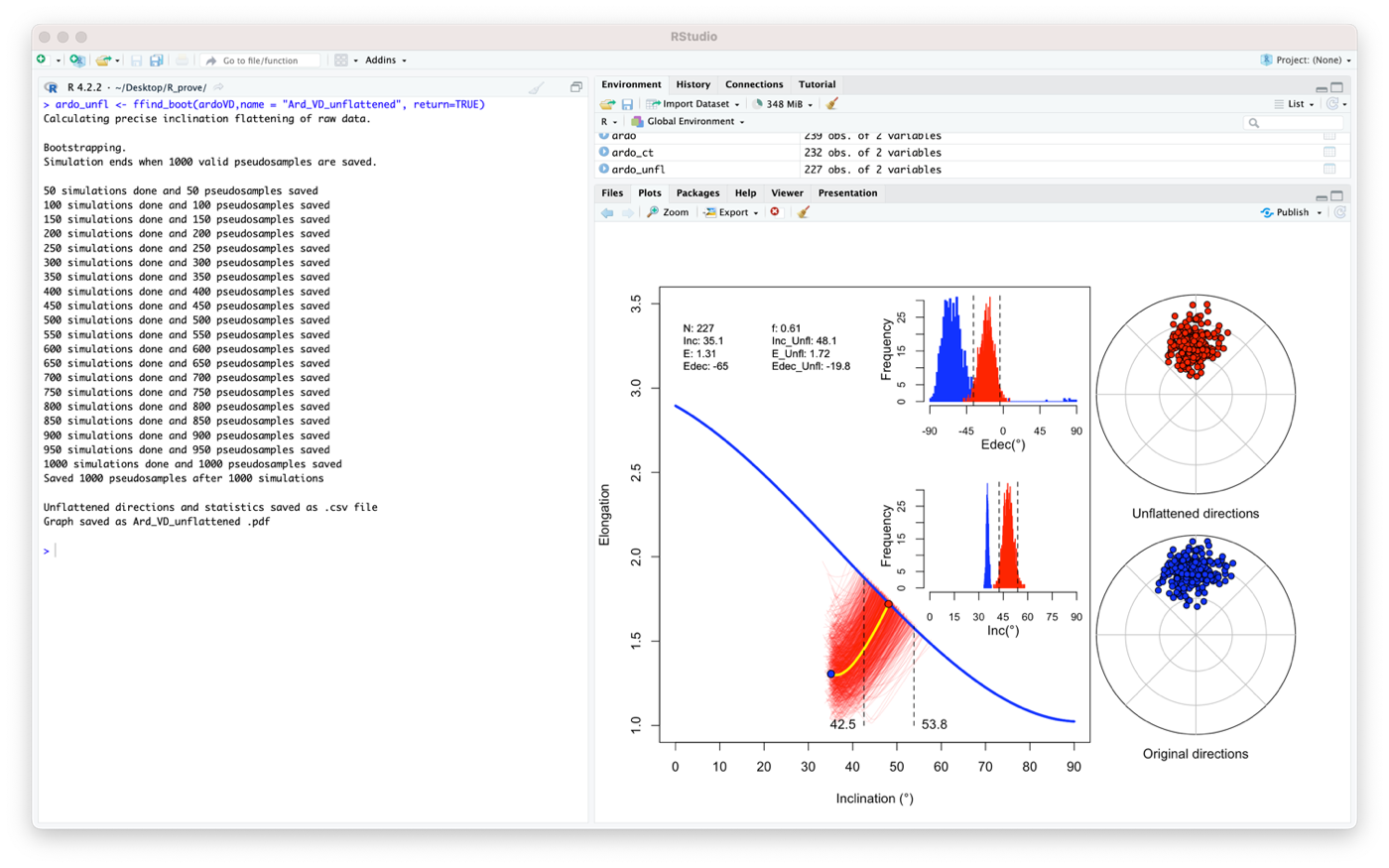


*Figure S8. Results of the elongation-inclination-based test*^5^ *for inclination flattening correction on the ardoVD dataset. Main result (yellow line ending and red dot on the crossing point with the expected elongation curve) are shown together with the same process applied on 1000 bootstrapped pseudosamples (red lines) and the derived 95% confidence boundaries. The two histograms show the inclination and elongation of declination of the bootstrapped datasets as occurring frequency. The original and unflattened datasets are shown in the equal area diagrams to the right.*

Next, we wish to plot the directions corrected for inclination flattening, and the average:

> plot_DI(ardo_unfl, symbol = "t", col_d = "brown",col_u = "yellow", title = "Ardo filtered and unflattened")

> fisher_plot(ardo_unfl, col_d = "dark green", col_u = “light green”, col_l = "red”, save = T, name = "Ardo_Unfl_fisher", text=T)

will return the plot of Figure S9.


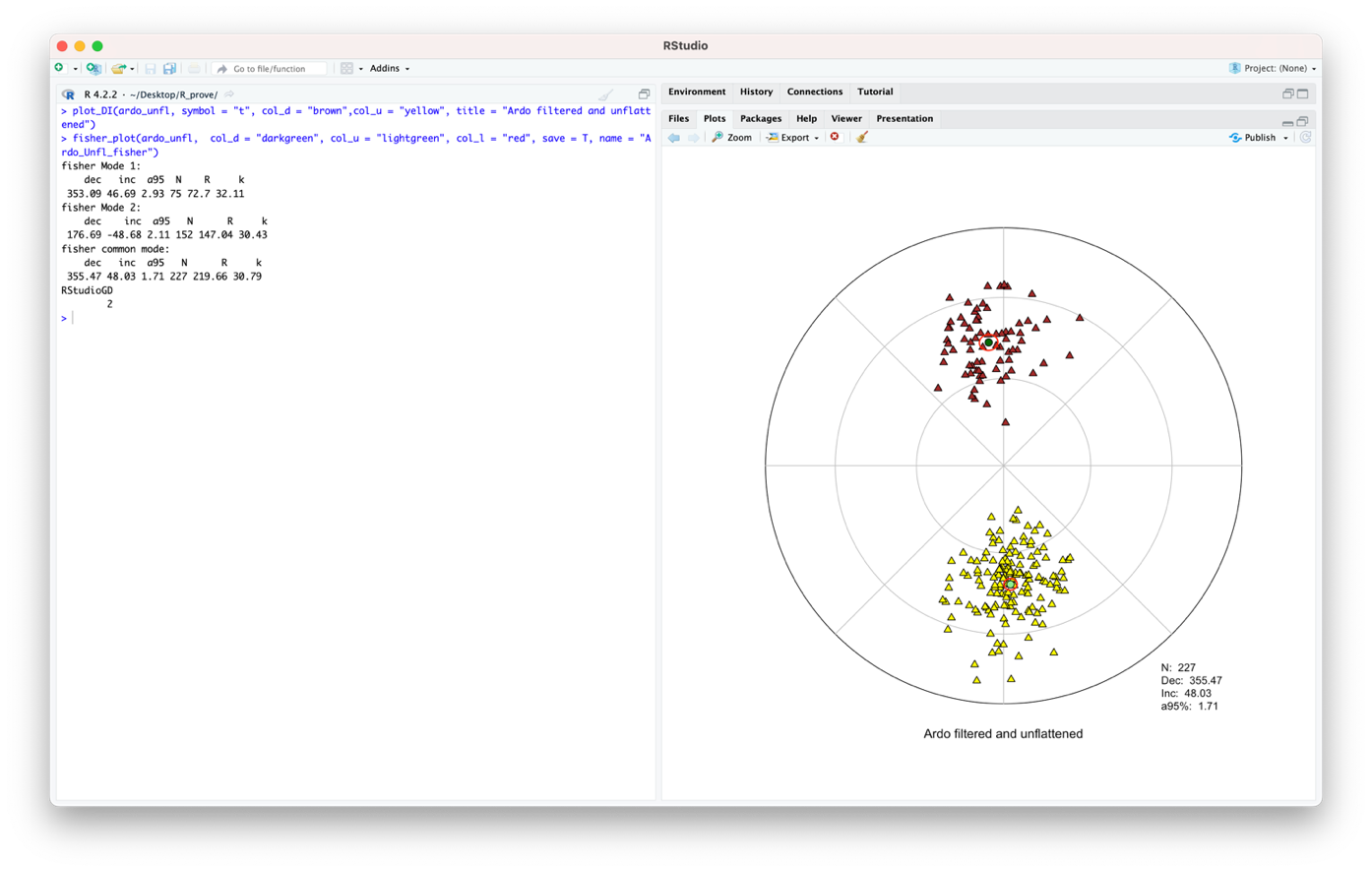


*Figure S9. Application of Fisher*^3^ *statistic on the filtered and unflattened Ardo_PmagDiR set of paleomagnetic directions, followed by the calculation of the virtual geomagnetic poles (VGPs) position and average paleomagnetic pole.*

**Plotting (virtual) geomagnetic poles and confidence**

If the results are satisfactory, the list of VGPs and the average paleomagnetic pole can be calculated. An example is:

> ardo_vgp <- VGP_DI(ardo_unfl, lat = 46.04,long = 12.14,type = "VGPsN", name = "Ardo_VGPs")

The function *PmagDiR::VGP_DI* automatically exports four csv text files within the working directory, which are called and described as follows:

- *name­*_average_pole.csv: paleomagnetic pole with associated Fisher^3^ statistical parameters.
- *name*­_bimodal.csv: virtual geomagnetic poles as resulted from calculation.
- *name_*rotated.csv: virtual geomagnetic poles rotated with the average pole coinciding with the spin axis (option for magnetic polarity reversals plotting).
- *name*_single_mode.csv: virtual geomagnetic poles in a single (northern hemisphere) mode.

The result file returned into the RStudio environment is defined by the command “*type*”, and in the current example is the single mode (VGPsN, which is also the default mode if type is not specified in the command line). The VGPs can be plotted onto a spherical orthographic (i.e., view from infinite point) projection by using the function *PmagDiR::plot_VGP.*

> plot_VGP(VGP = ardo_vgp, grid = 20, auto_cent = T, coast = T,A95 = T, name = "ardo_VGP")

The command “*grid*” allows to define the angular distance between the parallels and meridians. The longitude and latitude of the projection center can be defined within the command or set to automatically coincide with the paleomagnetic pole buy setting the auto centering command TRUE (*auto_cent= T*). The present-day coastlines can be added to the projection for clarity (coast= T), as well as the paleomagnetic pole calculation (A95= T) (Figure S10).


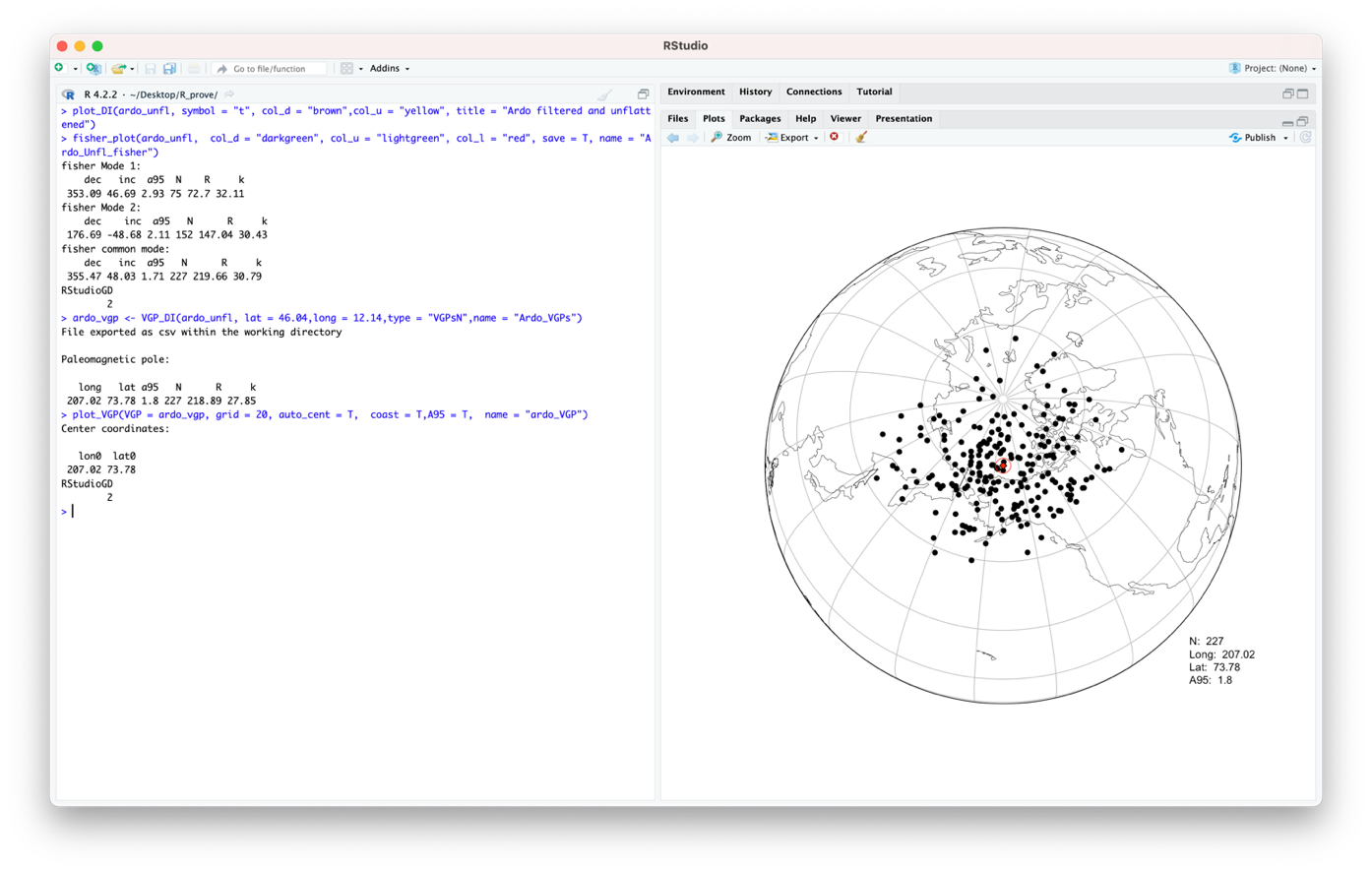


*Figure S10. Spherical orthogonal projection of the virtual geomagnetic poles (VGPs) and their average direction as calculated by the function PmagDiR::VGP_DI and plotted with PmagDiR::plot_VGP.*

The paleomagnetic pole can be plotted separately for comparison with the global synthetic apparent polar wander path (GAPWP^6,7^). The 95% confidence angle can be determined in two different ways: either by using standard Fisher^3^ or by calculating the average pole of a number (default is 1000) of bootstrapped pseudosamples^8^. In this case the angular distance of the bootstrapped poles with respect the real paleomagnetic pole is also plotted as a function of frequency, and the 95% confidence angle is defined by the nb*0.95^th^ (where nb= number of bootstrapped pseudosamples) ranked bootstrapped pseudosample mean. The command:

> VGP_boot(VGP = ardo_vgp,nb = 2000,auto_cent = T,grid = 20,coast = T,name = "VGP_boot")

returns the plot shown in Figure S11. By executing the command, once the bootstrapped process is concluded, both the *PmagDiR::VGP_boot* and the *PmagDiR::VGP_A95* functions give the possibility to compare the result with the GAPWP. By default, *PmagDiR* adopts the recent GAPWP of Vaes et al. (2023; V23_GAPWP)^7^, but the GAPWP of Torsvik et al., (2012; T12_GAPWP)^6^ can be plotted by selecting it in the function command (please refer to the help documentation). This is executed by direct interaction within the console, as shown in Figure S11. In this specific case, the VGP data from the *Ardo_PmagDiR* record (filtered for outliers and corrected for inclination flattening) are compared with the 20 Ma to 100 Ma GAPWP plotted in South African coordinates. The paleomagnetic pole (Paleocene) is virtually coinciding with the GAPWP between 50 and 60 Ma, as expected (Figure S11). In the same Figure (S2.11) the paleomagnetic pole of the original (no cut-off and no inclination flattening correction) *Ardo_PmagDiR* set is calculated and compared with the data as determined through all steps described above.

> ardo_vgp_flawed <- VGP_DI(DI = ardo, lat = 46.04, long = 12.14, export = F)

> VGP_boot(VGP = ardo_vgp_flawed, nb = 2000, lat = 73.78, long = 207.02, auto_cent = F, on_plot= T, color = "yellow")

The paleomagnetic pole is significantly departed from the reference GAPWP, despite the presence of reversals and positive antipodality test. This highlights the importance of an accurate evaluation of the paleomagnetic directions reliability.


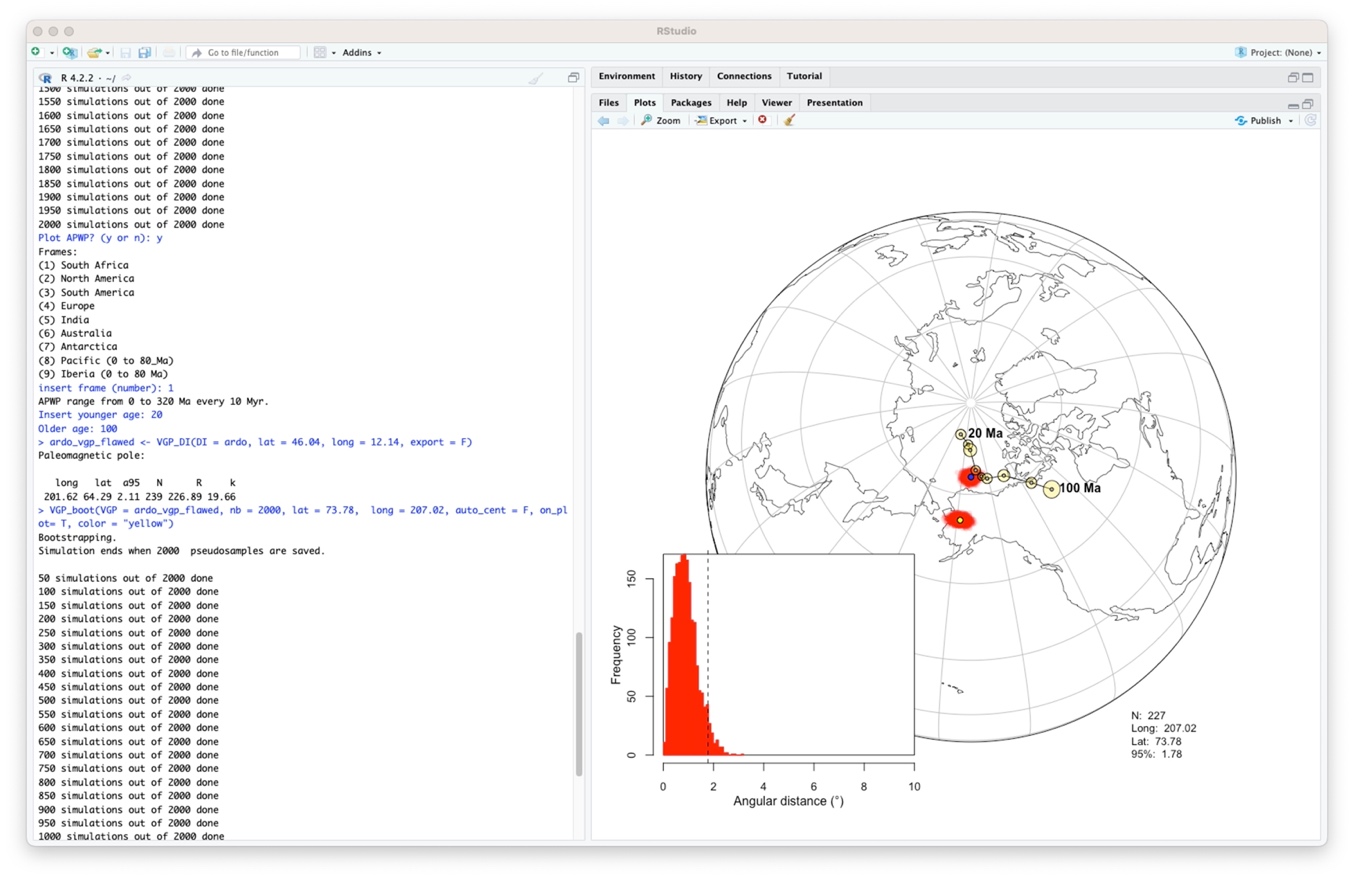


*Figure S11. Paleomagnetic calculated from the Ardo_PmagDiR dataset after filtering the data for outliers and correcting for paleomagnetic inclination shallowing (blue dot) and by using the original data without any treatment (yellow dot), shown with the bootstrapped confidence angles. The histogram shows the confidence angle calculation of the unflawed (blue) pole, specified numerically in the bottom-right hand of the panel. Data are shown with the reference global synthetic apparent polar wander path (GAPWP) between 20 Ma and 100 Ma in South African coordinates*^7^*.*

**Correct strain-biased paleomagnetic direction**

Dallanave et al. (2020)^2^ published a set of paleomagnetic directions from early–middle Eocene carbonate rocks exposed in northern New Caledonia near Koumac. The carbonate rocks record includes 83 stratigraphic meters of massive micrite from which have been isolated 88 paleomagnetic directions. Directions are organized in two modes statistically antipodal. Despite the primary origin of the remanence, comparison with coeval datasets indicates that the directions underwent some degree of deviation from the expected orientation^9^. Analysis of anisotropy of magnetic susceptibility (AMS) reveals an oblate magnetic fabric (k_1_≈k_2_>k_3_, where k_1,2,3_ are respectively the maximum, intermediate, and minimum axes of the AMS tensor) with the minor (k_3_) axis not perpendicular to the bedding plane, indicating the presence of pervasive strain (Figure 7 of the main text). If we consider the k_3_ axis parallel to the shortening direction^10^, in order to restore the original orientation of the paleomagnetic directions we should apply a prolate “unstrain” matrix (U) with the form u_1_>u_2_=u_3_ with u_1_ oriented parallel to the AMS k_3_ axis.

The first step is to calculate the eigenvector matrix (M) of the AMS inverse. The AMS matrix of the Koumac dataset is stored within *PmagDiR* as *km_AMS*, and can be visualized by typing in the console (Figure S12):

> km_AMS

It consists of one row and nine values, in the form of the AMS eigenvalue v_i_, v_i_ declination (v_i__dec), and v_i_ inclination (v_i__inc), with i= 1,2,3. To calculate M we can type:

> M <– AMS_inv(km_AMS, type = “v”)

The command *type* indicates the form in which the matrix to invert is expressed, as explained in the function documentation. The anisotropy parameters of the input AMS matrix are automatically displayed in the console, specifically the lineation (L=v_1_/v_2_), foliation, (F=v_2_/v_3_), and anisotropy degree (P=v_1_/v_3_). The matrix M can be visualized by typing “M” (Figure S12). The matrix M provides only the eigenvectors (i.e., the orientation of the “unstrain” matrix U), and it does not hold information on the degree of “unstrain” that we wish to apply. As mentioned above, we wish to apply a prolate (u_1_>u_2_=u_3_) matrix “U”. The matrix U will be determined by the matrix M and the diagonal matrix S holding the information about the “unstrain” targets (see details in the Computational background). These are set arbitrarily by choosing a target lineation (Lin= s_1_/s_2_) of, for example, 1.3 (relatively high value if compared to the AMS fabric), keeping the foliation (Fol= s_2_/s_3_) equal to one. The function also requires the number of “unstrain” steps (n) in between Lin= 1 and the target Lin= 1.3:

> km_unstr <– unstr_DI(km_PmagDiR, S_vec= M, Lin = 1.3, Fol = 1, n = 50)

This command gives the result shown in Figure S12. It is important that the paleomagnetic directions file (*km_PmagDiR* in this case) has four columns with declination, inclination, bedding dip azimuth, and bedding dip, because the unstrain protocol affects also the bedding. The *unstr_DI* command automatically export the unstrained directions in tilt corrected coordinates within the working directory, but the result file (*km_unstr* in this case) saved in the R environment consists of a complex list including many information, and it will be used to calculate the confidence boundaries. In order to explore the file, just type “km_unstr” in the console.


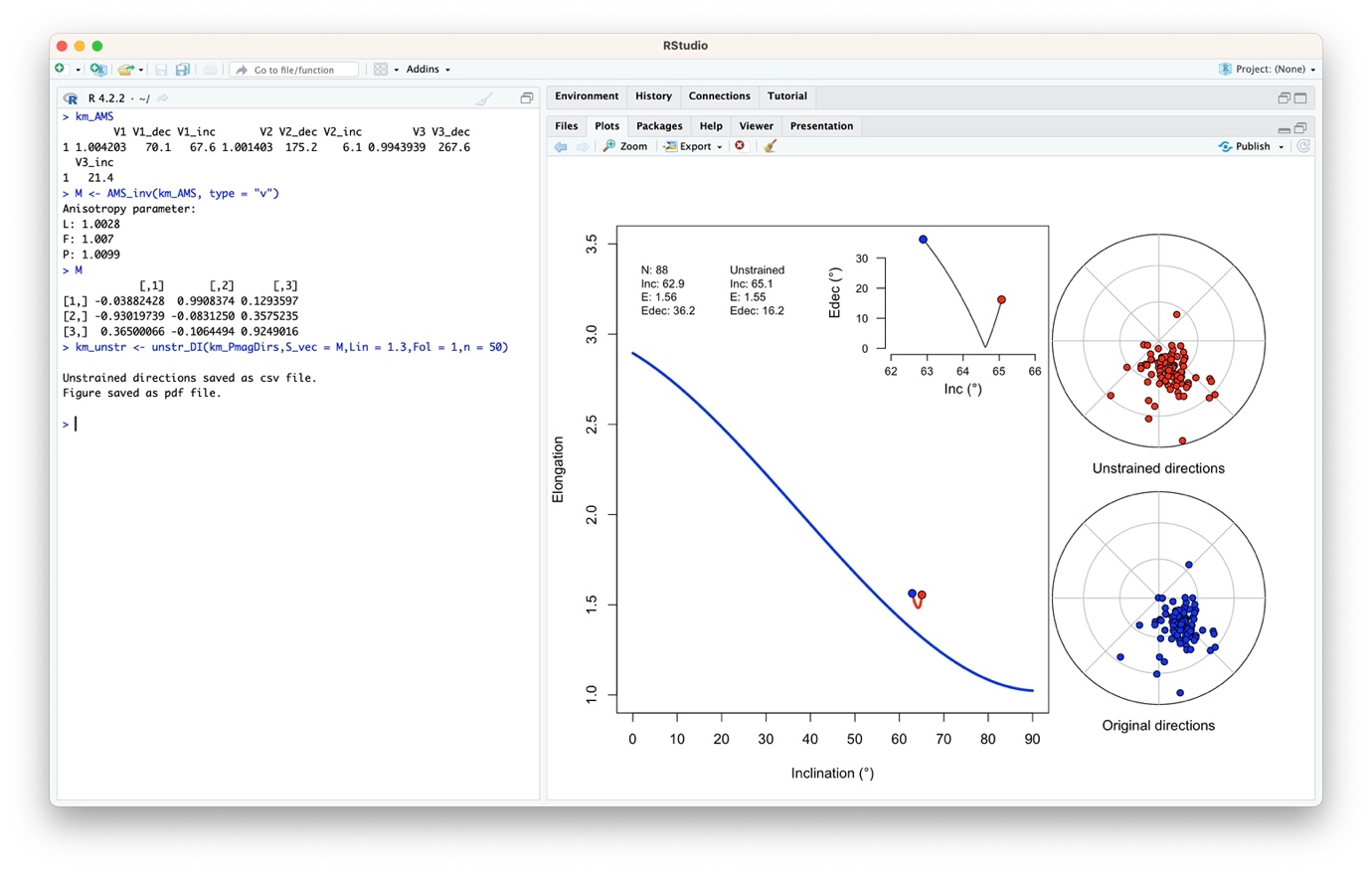


*Figure S12. First explorative “unstrain” process of the km_PmagDiR directions set. The process stops when the target lineation (Lin=1.3) is reached.*

As described in the main text, we observe that elongation-inclination path of the *km_PmagDiR* distribution do not cross the reference TK03.GAD line, but Edec (declination of elongation) reaches a minimum between 64° and 65° (Figure S12, inset in main figure). If we consider that minimum as the “unstrain” target, we can repeat the protocol setting the EdMIN=TRUE (Figure S13):

> km_unstr <– unstr_DI(km_PmagDiR, S_vec=M, Lin= 1.3, Fol=1,n=50, EdMIN=TRUE)

The algorithm now stops when Edec is minimalized.

**
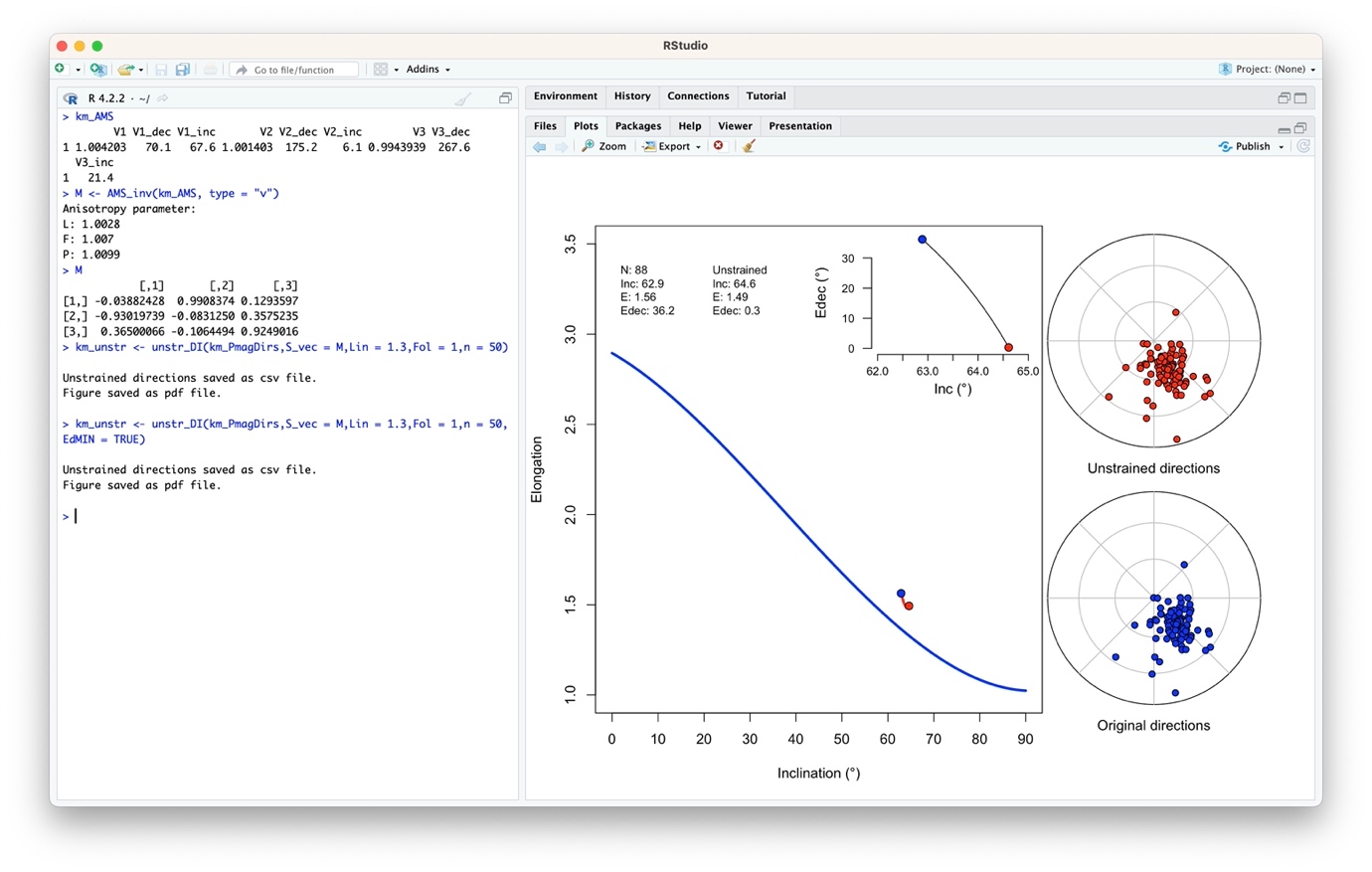
**

*Figure S13. Application of the second “unstrain” cycle, sets to interrupt when the declination of the elongation (Edec) is minimized (EdMIN=TRUE within the command line).*

Now we can calculate the angular confidence of the unstrained directions by applying the same protocol to a number of bootstrapped pseudosamples by using the command:

> unstr_boot(km_unstr, nb= 1000, S_vec=M, Lin=1.3, Fol= 1, ns=20, EdMIN=TRUE, hist=FALSE)

When calling the *unstr_boot* function, we must use the result file from the *unstr_DI* function, in this case called “km_unstr” (Figure S14). Among the options, “nb” is the number of bootstrapped pseudosamples, “ns” is the number of increments between the first reiteration and the target unstrain (equal to “n” of the *unstr_DI* function, not to be confused with “nb”). The option *hist*, if set TRUE, would plot the Edec histogram on the top right hand of the main plot analogously to Figure S8 (*ffind_boot* function).

**
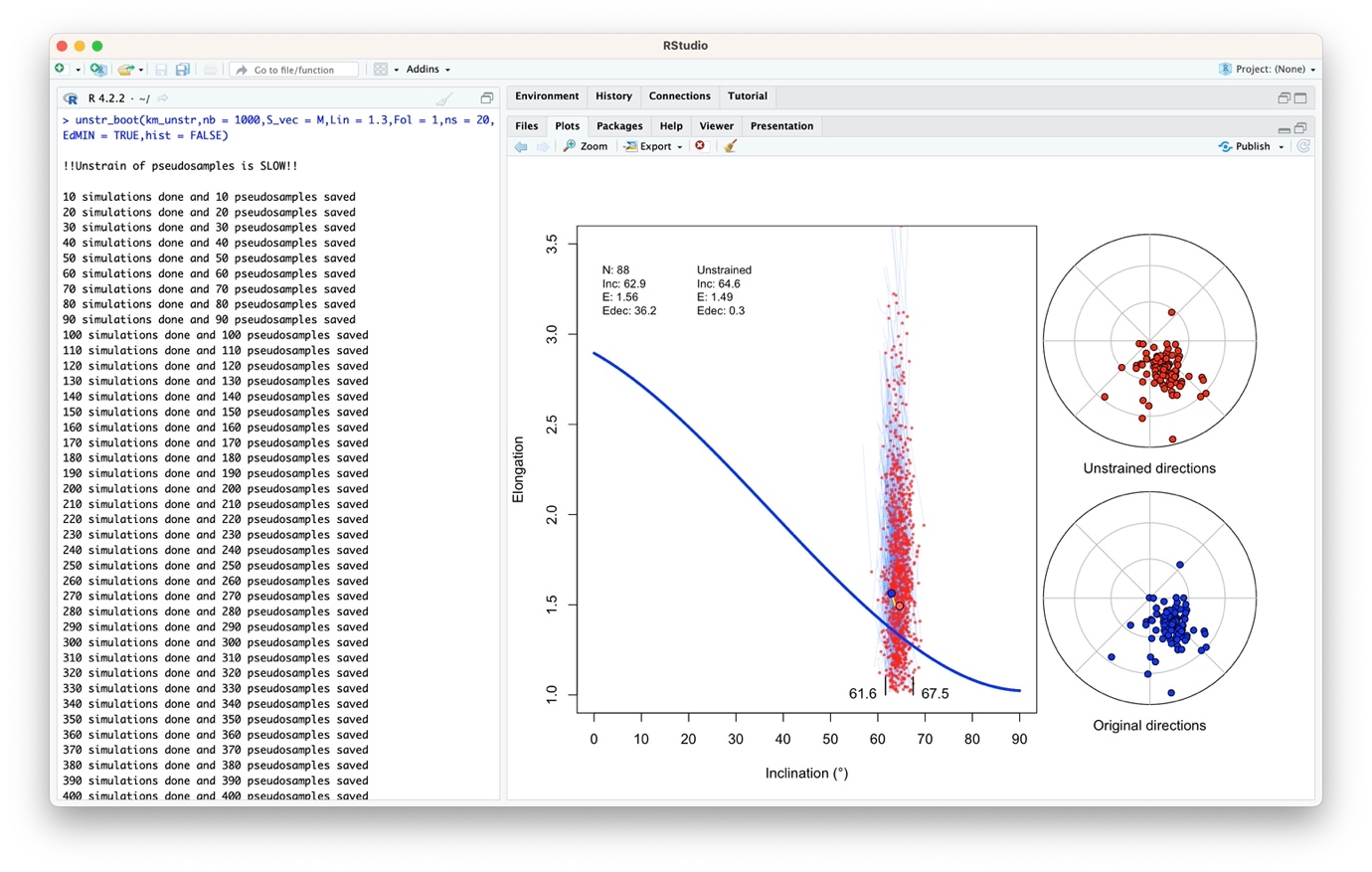
**

*Figure S14. The “unstrain” algorithm is repeated on 1000 bootstrapped pseudosamples.*

Because the unstrain process of the bootstrapped pseudosamples is relatively time demanding (tens of minutes on average), it is recommended to keep the ns value lower than the initial evaluation with *unstr_DI* (in our case, ns=20). At the end of the process, confidence boundaries and figure are automatically saved within the working folder.

**References**

1. Dallanave, E., Agnini, C., Muttoni, G. & Rio, D. Paleocene magneto-biostratigraphy and climate-controlled rock magnetism from the Belluno Basin, Tethys Ocean, Italy. *Palaeogeogr. Palaeoclimatol. Palaeoecol.* **337–338**, 130–142 (2012).

2. Dallanave, E. *et al.* Eocene (46-44 Ma) onset of Australia-Pacific plate motion in the southwest Pacific inferred from stratigraphy in New Caledonia and New Zealand. *Geochem. Geophys. Geosystems* **21**, (2020).

3. Fisher, R. Dispersion on a sphere. *Proc. R. Soc. Lond.* **A217**, 295–305 (1953).

4. Vandamme, D. A new method to determine paleosecular variation. *Phys. Earth Planet. Inter.* **85**, 131–142 (1994).

5. Tauxe, L. & Kent, D. V. A simplified statistical model for the geomagnetic field and the detection of shallow bias in paleomagnetic inclinations: Was the ancient magnetic field dipolar? in *Timescales of the Paleomagnetic Field, Geophys. Monogr.* (eds. Channell, J. E. T., Kent, D. V., Lowrie, W. & Meert, J. G.) vol. 145 101–115 (American Geophysical Union, 2004).

6. Torsvik, T. H. *et al.* Phanerozoic polar wander, palaeogeography and dynamics. *Earth-Sci. Rev.* **114**, 325–368 (2012).

7. Vaes, B. *et al.* A global apparent polar wander path for the last 320 Ma calculated from site-level paleomagnetic data. *Earth-Sci. Rev.* **245**, 1–35 (2023).

8. Tauxe, L., Kylstra, N. & Constable, C. Bootstrap statistics for paleomagnetic data. *J. Geophys. Res.* **96**, 11723–117490 (1991).

9. Dallanave, E. & Kirscher, U. Testing the reliability of sedimentary paleomagnetic datasets for paleogeographic reconstructions. *Front. Earth Sci. Geomagn. Paleomagn.* **8:592277**, 1–16 (2020).

10. Parés, J. M., van der Pluijm, B. A. & Dinarès-Turell, J. Evolution of magnetic fabrics during incipient deformation of mudrocks (Pyrenees, northern Spain). *Tectonophysics* **307**, 1–14 (1999).
